# Phylogenetic relationship and virulence inference of Streptococcus Anginosus Group: curated annotation and whole-genome comparative analysis support distinct species designation

**DOI:** 10.1186/1471-2164-14-895

**Published:** 2013-12-17

**Authors:** Adam B Olson, Heather Kent, Christopher D Sibley, Margot E Grinwis, Philip Mabon, Claude Ouellette, Shari Tyson, Morag Graham, Shaun D Tyler, Gary Van Domselaar, Michael G Surette, Cindi R Corbett

**Affiliations:** 1 National Microbiology Laboratory, Public Health Agency of Canada, Winnipeg, MB, Canada; 2 Department of Medical Microbiology, University of Manitoba, Winnipeg, MB, Canada; 3 Department of Microbiology, Immunology and Infectious Diseases, University of Calgary, Calgary, AB, Canada; 4 Departments of Medicine and Biochemistry and Biomedical Sciences, McMaster University, Hamilton, ON, Canada

**Keywords:** Streptococcus Milleri group, Streptococcus Anginosus group, *Streptococcus anginosus*, *Streptococcus constellatus*, *Streptococcus intermedius*, Phylogenetics, Virulence, Comparative genomics, Whole-genome sequencing

## Abstract

**Background:**

The Streptococcus Anginosus Group (SAG) represents three closely related species of the viridans group streptococci recognized as commensal bacteria of the oral, gastrointestinal and urogenital tracts. The SAG also cause severe invasive infections, and are pathogens during cystic fibrosis (CF) pulmonary exacerbation. Little genomic information or description of virulence mechanisms is currently available for SAG. We conducted intra and inter species whole-genome comparative analyses with 59 publically available *Streptococcus* genomes and seven in-house closed high quality finished SAG genomes; *S. constellatus* (3), *S. intermedius* (2), and *S. anginosus* (2). For each SAG species, we sequenced at least one numerically dominant strain from CF airways recovered during acute exacerbation and an invasive, non-lung isolate. We also evaluated microevolution that occurred within two isolates that were cultured from one individual one year apart.

**Results:**

The SAG genomes were most closely related to *S. gordonii* and *S. sanguinis*, based on shared orthologs and harbor a similar number of proteins within each COG category as other *Streptococcus* species. Numerous characterized streptococcus virulence factor homologs were identified within the SAG genomes including; adherence, invasion, spreading factors, LPxTG cell wall proteins, and two component histidine kinases known to be involved in virulence gene regulation. Mobile elements, primarily integrative conjugative elements and bacteriophage, account for greater than 10% of the SAG genomes. *S. anginosus* was the most variable species sequenced in this study, yielding both the smallest and the largest SAG genomes containing multiple genomic rearrangements, insertions and deletions. In contrast, within the *S. constellatus* and *S. intermedius* species, there was extensive continuous synteny, with only slight differences in genome size between strains. Within *S. constellatus* we were able to determine important SNPs and changes in VNTR numbers that occurred over the course of one year.

**Conclusions:**

The comparative genomic analysis of the SAG clarifies the phylogenetics of these bacteria and supports the distinct species classification. Numerous potential virulence determinants were identified and provide a foundation for further studies into SAG pathogenesis. Furthermore, the data may be used to enable the development of rapid diagnostic assays and therapeutics for these pathogens.

## Background

The genus *Streptococcus* consists of Gram-positive cocci that are divided into sub-groups via numerous biochemical and molecular methods. The majority of *Streptococcus* species can be divided into either β-hemolytic causing complete zones of lysis on blood agar plates or α-hemolytic, formation of green zones due to oxidation of hemoglobin by hydrogen peroxide to form a green methemoglobin. Lancefield typing (based on specific carbohydrates within the bacterial cell wall) provides groupings that do not necessarily follow recognized species. The most clinically important are *S. pyogenes* known as Lancefield Group A *Streptococcus* (GAS), *S. agalactiae* Lancefield Group B *Streptococcus* (GBS), Lancefield group O (*S. pneumoniae, S. mitis* and *S. pseudopneumoniae*) and the variable Lancefield group species belonging to the Streptococcus Anginosus group (Which has also been referred to as the Streptococcus Milleri Group primarily by clinicians; SAG) that include non-typeable (using Lancefield typing) as well as strains that are Lancefield group C, F and G. The majority of α-hemolytic streptococci are non-pyogenic including the viridans group *Streptococcus* (VGS = Anginosus, Mitis and Salivarius groups), Mutans, and *S. suis*, which is a species that has not been assigned to a group [1]. VGS are considered to be part of the normal microbiota in the human oropharyngeal, urogenital and gastrointestinal tracts [2]. Many of the VGS are classified as α-hemolytic based on their activity on standard sheep’s blood agar. However, some strains can be β-hemolytic, including *S. anginosus* and *S. constellatus* that show beta-hemolytic activity and have been shown to produce a streptolysin S-like protein [3]. *S. intermedius* produces beta-hemolysis on human blood due to a human specific hemolysin called intermedilysin [4] and may also be beta-hemolytic on sheep blood agar. Many other VGS behave similarly and also show β-hemolytic activity under anaerobic conditions, but not aerobically under which these assays are usually conducted (Surette and Teal, unpublished data).

The taxonomic grouping of the SAG has historically been debated and the definitions have ranged from that of one to three species (with or without subspecies) [5]. The validity of *S. anginosus* (SA), *S. intermedius* (SI) and *S. constellatus* (SC) as individual species has been addressed through phenotypic analysis, DNA: DNA hybridization studies and genetic characterization, and currently there is little debate that there are at least three distinct species with additional subspecies [6,7]. A recent study has elucidated that SAG consist of 3 species with *S. constellatus* divided into 3 subspecies (subsp constellatus (SCC), subsp pharyngis (SCP) and subsp viborgensis) and *S. anginosus* divided into 2 subspecies [subsp anginosus (SAA) and subsp whileyi (SAW)], based on the use of seven core housekeeping genes [7]. The SAG are phenotypically diverse but most strains share common characteristics such as slow growth rate, distinctive ‘caramel smell’, ability to hydrolyze arginine, acetoin production from glucose, and an inability to ferment sorbitol [8]. Lancefield sero-grouping is variable with SA Lancefield types of A, C, F or G, while SC is typically Lancefield C, F or no antigen, and SI is generally not typeable using the Lancefield method. Almost half of all human SAG clinical isolates are Lancefield F type [9]. Due to this phenotypic variability, molecular methods must be used for proper classification of SAG.

The SAG are part of the microbiota of the respiratory, gastrointestinal, and genitourinary tract with variable carriage levels [8]. The SAG are also medically important for their ability to cause suppurative infections and have been isolated from numerous body sites [10,11]. Of particular interest, SAG has been identified as the most common organism isolated from brain abscess [12,13], liver abscess [14] and empyema [13,15]. Their capacity to elicit pulmonary exacerbation and contribute to disease pathology in CF has also been demonstrated [14,16,17]. However, the exact mechanism for virulence within SAG has yet to be determined.

Although many *Streptococcus* species have the ability to cause disease, virulence studies within the *Streptococcus* genus often focus on GAS, GBS and *S. pneumoniae*[18-20]. SAG virulence and pathogenesis mechanisms have not yet been well studied; however, virulence mechanisms have been identified within SAG that allow for the invasion of host cells, evasion of host immune activity, spreading, and allow for the colonization of host tissues to occur. Intermedilysin is a cholesterol-dependent cytolysin produced by all SI strains that demonstrates specificity for human erythrocytes [21], and is essential for invasion of human cells by SI [4]. *S. constellatus* and SA also exhibit the β-hemolytic phenotype on sheep’s blood agar [22], this hemolytic activity has been attributed to the cytolytic factor Streptolysin S-like peptides encoded by the *sag*A gene within a *sag* operon [23]. Mutation to the *lux*S gene has also been shown to decrease hemolytic activity in SI [23]. Capsules allow evasion of the host immune system and encapsulated SAG strains have been isolated having a greater virulence potential than non-encapsulated strains [24]. They are more likely to cause larger abscesses, earlier spontaneous drainage and death in mice compared to non-encapsulated strains [25]. Hyaluronan (HA) is a major component of the extra cellular matrix of human connective tissue and is expressed by many cell types [26]. HA up regulates the *hyl* gene increasing SAG spreading and colonization within the host [27]. Most SAG isolates have both hyaluronidase and chondroitin sulfatase activity [9]. A detailed analysis of SAG virulence targets are required to achieve a firm understanding for the overall virulence potential within SAG.

The number of sequenced bacterial genomes has exploded with the advent of new sequencing technologies, which has allowed for comparative genomic analysis. Using Roche GS20 and Illumina technologies, we have sequenced to closure and, sequence polished and fully annotated seven SAG genomes including representatives of each species. With the abundance of sequenced strains within the *Streptococcus* genus, we conducted a detailed comparative analysis utilizing 66 streptococcal genomes including representatives from SAG, Mitis, Pyogenic, Salivarius, Bovis, Mutans groups and *S. suis* strains. Such genomic comparisons allowed for detailed characterization with insights gained into SAG phylogenomics, core genome, virulence potential, horizontally transferred genetic material, and microevolution within the host.

## Results and discussion

### An introduction to the SAG genome structure

The SAG strains were chosen for study based on being the numerically dominant bacterial isolate cultured during CF pulmonary exacerbation or primary culture from an invasive infection (Table 1). All strains were sequenced using Roche GS20 and Illumina sequencing technologies. The sequence coverage for each genome can be observed in Additional file 1. All seven genomes were sequenced to full closure with additional Sanger and Illumina sequence polishing to correct sequence errors and false pseudogenes. Polishing was used to correct homopolymer base-calling errors known to occur with the GS20 pyrosequencing reactions. Illumina sequencing was not done for SCP C232 as Sanger sequencing was done previously to evaluate homopolymer errors and achieve genome closure.

**Table 1:** Background information for SAG strains used in this study

**Species**	**Strain**	**Lancefield type**	**Case**^**a**^	**Extent of SAG disease**	**Clinical outcome**	**Genotype**	**Age/Sex**
SA	C238	C	3	Recurrent broncho-pulmonary	Transplant	∆F508/∆F508	18/F
SA	C1051	C	NA	Blood sepsis	NA	Non-CF	NA
SCP	C232	C	4	Recurrent broncho-pulmonary	Stabilized	∆F508/∆F508	30/M
SCP	C818*	C	4	Recurrent broncho-pulmonary	Stabilized	∆F508/∆F508	31/M
SCP	C1050	C	NA	Blood sepsis	NA	Non-CF	NA
SI	C270	NT	5	Broncho-pulmonary	Stabilized	∆F508/∆F508	32/F
SI	B196	NT	1	Broncho-pulmonary, septic arthritis, Osteomyelitis, pyomyositis	Total hip arthroplasy	∆F508/∆F508	18/M

^a^Case history as described in Parkins et al. [17]; *SCP C818 was isolated from the same individual 13 months after SCP C232; NT, Non-typable; NA, Not available for these isolates.

**Figure 1:**
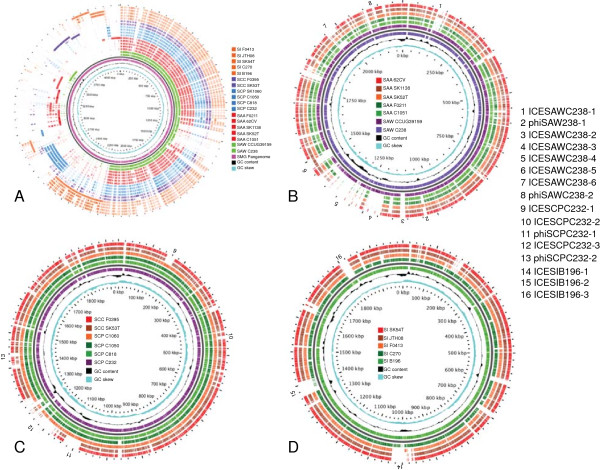
**Schematic view of the Pan-genome and the circular SAG genomes based on nucleotide identity of >70%.** An overall pan-genome Figure was constructed as well as figures for each species within SAG. The figures are arranged as follows; **A)** Pan-genome comparison of SAG; the genomes were constructed by adding sequences unique to the pangenome at the end of the chromosome it is found in; **B)** Circular genome comparison of SA with SAW C238 as the reference genome; **C)** Circular genome comparison of SC with SCP C232 as reference genome; and **D)** Circular genome comparison of SI with B196 as the reference genome; For all diagrams the order of rings from innermost to outermost is: the ruler in kbp, the G + C skew (purple ring) with positive strand >0, and negative strands <0; G + C content positive strand >0 and negative strand <0 (black ring), followed by the genomes as listed in the legends. The numbers on the outer rings indicate regions of horizontal gene transfer including ICE; Integrative conjugative element and phi; bacteriophage regions.

Each finished genome of the seven in-house sequenced SAG was comprised of a single circular chromosome ranging in size from 1.91 Mbp to 2.23 Mbp and is within the range of genome sizes observed for all available sequenced SAG strains (Table 2). A schematic view of all sequenced SAG genomes, including the draft genome sequences available from NCBI are provided in Figure 1, with an overall SAG comparison showing the pan-genome in Figure 1A and a comparison of each species in Figure 1B-D. The average genome size for SAG is very similar to the 2.02 Mbp average among the streptococci used for this study (*n* = 66). An overall comparison, using MAUVE, of the different species within SAG revealed that SI and SC had long stretches of synteny with regions of rearrangement. SA had a greater amount of variation, although 11 of the genomes were draft genomes (contigs) meaning that synteny is difficult to infer (Additional file 2). The BLAST atlas comparison for Figure 1B-D allows for a comparison of all strains to a reference strain for each species of SAG, and reveals regions of divergence within the genomes relative to the reference strain. The general features of the SAG genomes are summarized in Table 2, and an overall summary of all genomes used in this study with publicly available genomic sequence data (Additional file 3). As previously identified SI F0395 was incorrectly submitted as an SI and has subsequently been shown to be a SCC [7].

**Table 2:** Summary of genome characteristics for sequenced SAG

**Species**	**Sub-species**	**Strain**	**Genome size**	**G + C%**	**CDSs**	**tRNA**	**Avg length CDS (nt)**	**Coding%**	**Pseudogenes**	**GenBank accession**
**SA**	**Anginosus**	**C1051**	**1,911,706**	**38.97**	**1728**	**58**	**941**	**85.04**	**40**	**CP003860**
**SA**	**Whileyi**	**C238**	**2,233,640**	**38.23**	**1976**	**58**	**945**	**83.55**	**80**	**CP003861**
SA	Whileyi	CCUG39159^T^	2,294,730	38.50	2146	40	930	86.58	0*	GCA_000257765
SA	Anginosus	SK1138	1,958,191	38.61	1852	33	943	88.36	0	GCA_000287595
SA	Anginosus	SK52^T^	1,892,386	38.65	1805	31	921	88.36	0	GCA_000214555
SA	Anginosus	F0211	1,993,790	38.44	1844	31	956	87.63	0	GCA_000184365
SA	Anginosus	62CV	1,816,149	38.80	1751	61	912	87.95	0	GCA_000186545
**SC**	**Pharyngis**	**C232**	**1,935,414**	**38.18**	**1756**	**59**	**916**	**83.12**	**69**	**CP003800**
**SC**	**Pharyngis**	**C818**	**1,935,662**	**38.18**	**1754**	**59**	**916**	**83.02**	**71**	**CP003840**
**SC**	**Pharyngis**	**C1050**	**1,991,156**	**38.13**	**1797**	**59**	**921**	**83.27**	**73**	**CP003859**
SC	Pharyngis	SK1060^T^	1,963,771	37.99	2186	50	754	83.45	0	GCA_000223295
SC	Constellatus	SK53^T^	1,840,061	37.92	1730	32	943	87.83	0	GCA_000257785
SC	Constellatus	F0395	1,927,278	37.94	1861	61	916	87.63	0	GCA_000234015
**SI**		**C270**	**1,960,728**	**37.65**	**1778**	**60**	**949**	**86.09**	**24**	**CP003858**
**SI**		**B196**	**1,996,214**	**37.56**	**1815**	**60**	**952**	**86.53**	**18**	**CP003857**
SI		SK54^T^	1,919,718	37.58	1778	28	949	87.49	0	GCA_000258445
SI		JTH08	1,933,610	37.71	1780	67	979	86.59	0	AP010969
SI		F0413	1,921,346	37.63	1820	62	925	87.02	0	GCA_000234035

*There are no genes annotated as pseudogenes, however, there are genes that are annotated with potential sequence errors or frameshift mutations. Strains that have been bolded are high quality finished genomes from this study. T: denotes reference strain, SA: *Streptococcus anginosus*, SC: *Streptococcus constellatus*, SI: *Streptococcus intermedius.*

The G + C content for SAG strains ranged from 37.56 to 38.97% (avg 38.14, *n* = 18), with the overall G + C content increasing from SI, SC to SA (Table 2). Overall the average of 38.14% is similar to the 38.57% average G + C content for all *Streptococcus* strains analyzed (Additional file 3). None of the SAG strains contained plasmid DNA similar to other *Streptococcus*[28-30]. All seven in-house sequenced SAG contained four rRNA operons and 58, 59 and 60 tRNA genes in SA, SC and SI respectively, with the majority of tRNA genes situated around rRNA operons, as seen with other sequenced *Streptococcus* strains [31]. With regard to the number of tRNA and rRNA found within the finished genomes, SAG were most similar to *S. mitis* and *S. suis*[30], having fewer numbers of both these RNA genes than streptococci in the pyogenic, mutans, bovis and salivarius groups [28,29,32-35].

The SAG strains had on average 1800 CDSs (coding DNA sequence: does not include pseudogenes) with an average length of 934 bp, while the streptococcal average was 1943 CDSs with an average size of 893 bp. This difference in number of CDSs may be due in part to the differing size of streptococci genomes, but it may also be partially due to the extra care taken to manually annotate the in-house SAG genomes resulting in a higher number of pseudogenes. This has been demonstrated using *S. pyogenes* MGAS315, MGAS8232, SF370 and SSI-1, which in the original annotation had zero pseudogenes [29,32-34], but when retrospectively examined for the presence of pseudogenes they were shown to have 42, 50, 60 and 51 respectively [36]. The seven in-house sequenced SAG genomes harbor 16 to 80 pseudogenes depending on the strain, with the most found in SAW C238 and the least in SI B196 (Table 2). When pseudogenes are included in the total CDSs, the average number of CDSs increased to 1855. An example of how sequence errors or pseudogenes can affect the average CDS size is seen in the whole-genome shotgun sequence for SCP SK1060. This genome had an average CDS size of 754 bp (Table 2), however, a closer look at this genome revealed a large number of essential genes that were truncated including, *par*C, *gyr*A, *rpo*A, *uvr*A, *dna*G, *dna*E, and *fts*A. The lack of extra genetic material as shown in Figure 1A for SCP SK1060 compared to other SC strains also shows that the extra CDSs are created owing to sequence errors and not novel genetic materials. In this study, even after manual sequence verification, generating high quality finished genomes, all seven in-house sequenced SAG strains still contained predicted pseudogenes, defined as a full-length CDS present within another SAG strain from this study or GenBank or another genome in GenBank. This demonstrates the value of having single contig, high quality reference genomes for SAG to aid in the analysis of comparative genomic studies.

### Accurate Phylogeny of SAG requires multiple genetic loci

With the increased genomic sequences available, the capacity for applying phylogenetic analysis methods has increased; however, there is urgent need to evaluate their equivalency. Phylogenetic analysis using the single locus 16S rRNA has historically been the primary molecular method determining species within the SAG and, indeed this is how the seven in-house sequenced SAG strains were originally speciated. To assess the discriminatory power of 16S rRNA sequencing we compared the results to those acquired from an in-house core-SNP pipeline, a multi-locus automated pipeline for phylogenomic analysis called AMPHORA [37], and two alternate single loci targets *cpn*60 and *rpo*B. For all analysis strategies, 66-sequenced streptococcal genomes were included in the SAG clustering analysis. In all iterations SI*,* SC*,* and SA strains clustered together by species with one exception-SA F0211 clustered with the SC strains when using *cpn*60 as a reference.

**Figure 2:**
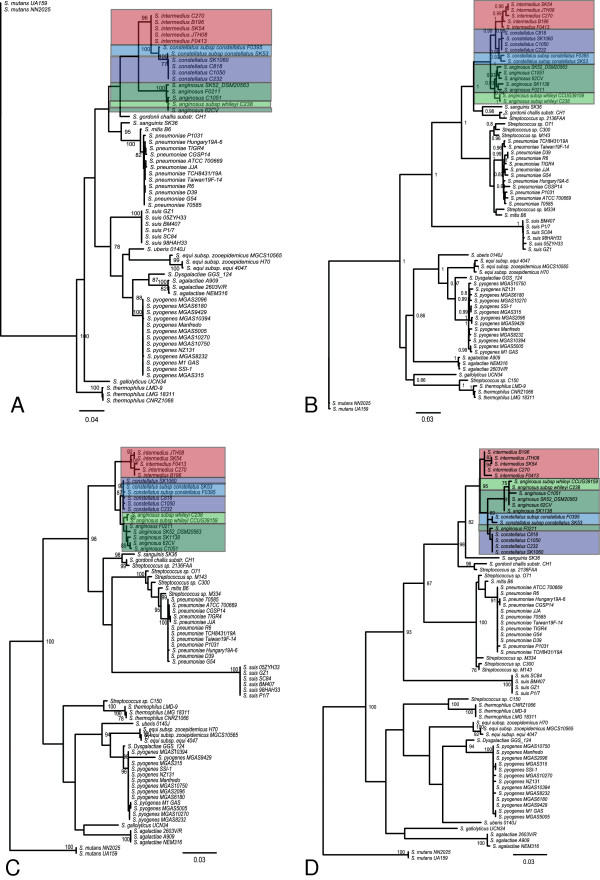
**Phylogenetic relationship among *Streptococcus *strains.** Four phylogenetic trees were constructed, each using a different genetic locus or multiple loci for analysis as follows: **A)** 16S rRNA, **B)** In-house core-SNP pipeline, **C)***rpo*B, and **D)***cpn*60. Bootstrap values of equal or greater than 75%,or approximate maximum likelihood ratio test values equal to or greater than 0.8 are shown. The blue boxes highlight SCC, purples boxes highlight SCP, the red boxes highlight SI, dark green boxes highlight SAA and light green highlights SAW*.*

Compared to the dendrogram constructed for 16S rRNA (Figure 2A), dendrograms of similar overall topology were acquired for all methods (Figure 2B, core-SNP pipeline; 2C, *rpo*B and 2D, *cpn*60) [Amphora (not shown)]; in general the branch lengths were longer with the multi-locus approaches and a greater degree of statistical certainty was achieved. It has been suggested that *rpo*B is a sensitive single locus method for differentiating Streptococcus. Housekeeping genes, such as *rpo*B and *cpn*60 are believed to be either selectively neutral or subject to purifying selection. The rate of synonymous substitutions (dS) should be equal to or slightly greater than the rate of nonsynonymous substitutions (dN), giving a dN : dS ratio of less than 1. The ratio of mean dN : dS per site (dN:dS ratio) for *rpo*B was found to be 0.106 and the ratio for *cpn*60 was 0.056 indicating purifying selection and demonstrating their suitability for use in phylogenetic analysis. Our analysis revealed *cpn*60 to provide the longest branch lengths discriminating one strain from another (Figure 2D), thus outperforming both *rpo*B (Figure 2C) and 16S rRNA (Figure 2A), supporting the findings of Glazunova (2006) [38]. For all analytic approaches, except for 16S rRNA, the closest related species to the SAG were *S. sanguinis* and *S. gordonii*. These cluster in a tight group away from all other sequenced streptococcal strains as previously demonstrated [7]. Schouls (2003) have previously demonstrated that speciation based on 16S rRNA is problematic given there is demonstrated lateral transfer within the SAG of portions of the 16S rRNA gene [39]; our study supports this fundamental limitation of 16S rRNA as a phylogenetic marker and demonstrates that 16S rRNA has lower discriminatory power for speciation than any of the other approaches tested herein. Although *cpn*60 and *rpo*B each performed reasonably well for SAG phylogenetic discrimination, we advocate that single locus results will be less reliable than results achieved with multiple genetic loci.

### Global ortholog analysis for SAG within the genus *Streptococcus*

A total genetic content comparison was conducted to determine the *Streptococcus* pan- and core genomes via ortholog comparison between 66 *Streptococcus* reference strains using OrthoMCL [40]. A total of 11587 orthologous groups were identified within the 66 *Streptococcus* strains, with 7669 ortholog groups absent within SAG (Additional file 4). In a similar study using 11 species and 45 strains of *Streptococcus*, 9053 orthologous groups were identified with 7442 absent within the *S. dysgalactiae* group [41]. The differences in numbers are due to an increased number of species (16) and strains (66) used in the present study, which is the largest study of this type done for *Streptococcus*. The *Streptococcus* pan-genome increases by an average of 45 genes for each of the 21 additional strains used [41]. Most genes are from the five new *Streptococcus* species generated in this study. This shows that the *Streptococcus* pan-genome should still be considered ‘open’ with new genes added with additional genomes analyzed.

**Figure 3:**
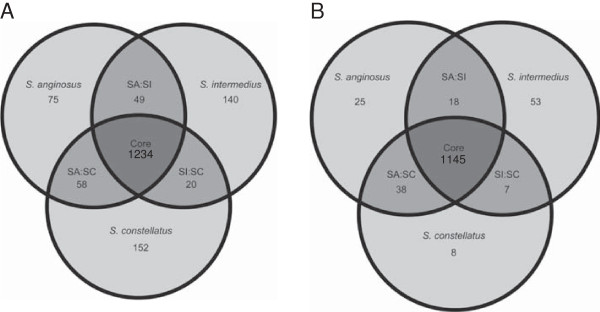
**Genomic content analysis of the sequenced SAG strains. A)** Comparison of seven SAG strains sequenced in this study showing the overall core genomes. **B)** Comparison of all available sequenced SAG strains. Numbers within circles correspond to number of CDSs that are conserved within the strains analyzed (intersection of strain circles) or that are unique to each strain analyzed.

The core genome of *Streptococcus* contains 626 genes, accounting for 32.2% (626/1943 (average genome #CDS) × 100) of the *Streptococcus* genome. Previous analysis suggests that the core genome should account for >25% of the average genome [42]. The core genome common to all seven in-house sequenced SAG was 1234 genes (Figure 3A), and decreased to 1145 when the 11 SAG draft genomes (WGS contigs) available in GenBank are added to the analysis (Figure 3B). The three comparator groups of pyogenic *Streptococcus* including Group A *Streptococcus* (GAS; *S. pyogenes*), Group B *Streptococcus* (GBS; *S. agalactiae*) and Group C *Streptococcus* (GCS; *S. equi*) have 1285, 1305 and 1325 core genes respectively [32]. The *S. dysgalactiae* group has 1471 genes common to both *S. dysgalactiae* subsp. *dysgalactiae* and *S. dysgalactiae* subsp. *equisimilis*[41]. The average core genome percentage for SAG is lower than other groups at only 62.2% (1145/1842 n = 18), however, it is more than the 60% threshold suggested as a limit for core genomes within streptococcal groups [42]. The majority of groups analyzed were for a single species rather than multiple species as done for SAG.

Comparison of individual species within SAG revealed that SC had the most core genes (1617) followed by SA (1323) and SI (1316), this is consistent with other species of streptococcus; *S. thermophilus* (1271) [43]; *S. pneumoniae* (1619) [44]; and *S. agalactiae* (1806) [45]. Within SAG the average percentage of core genes is 75.23% for SC, 70.7% for SA and 72.9% for SI*.* The values for SAG are similar to those found for other *Streptococcus* species including *S. pyogenes* (80.3%), *S. agalactiae* (79.1%) and *S. thermophilus* (81.4%) [42].

Extended analysis demonstrates that SAG shares the most core genes with *S. gordonii* and *S. sanguinis* (Table 3; Additional file 5). *S. anginosus* shares a slightly greater percentage of their core genome with *S. sanguinis*, whereas SI and SC share a very similar percentage of their core genome with both *S. gordonii* and *S. sanguinis* (Additional file 6). The finding that *S. gordonii* and *S. sanguinis* were most closely related to the SAG strains confirms previous studies [38]. Genome comparisons also confirmed that SAG shared a decreased portion of their core genome with less taxonomically related *Streptococcus* species including *S. pneumoniae*, *S. pyogenes*, and *S. agalactiae* (Table 3). The greatest numbers of orthologs from the core genome were found in *S. gordonii* (1073) and *S. sanguinis* SK36 (1067), while the fewest were found in *S. pyogenes* with 887 (Table 3, Additional file 5). This is partly due to the closer taxonomic relationship between SAG, *S. sanguinis* and *S. gordonii*, however, it may also be attributed to the number of genomes used in the analysis, with groups that have a greater number of sequenced genomes having a fewer number of core gene orthologs. Interestingly, *S. mutans* (which is less related to the SAG based on taxonomic analysis) shared more genes than some of the more closely related strains (Table 3, Additional file 5). This was also seen in a previous study that showed *S. sanguinis* shared more genes with *S. mutans* than the more closely related *S. pneumoniae*[31]. The most plausible explanation is the shared ecological niche that they all inhabit, however, *S. pneumoniae* is known to have increased virulence relative to *S. sanguinis* and *S. mutans*.

**Table 3:** **Comparison of gene content of SAG to clinically important *Streptococcus *species**

**Species**	**Average Core genes**	**# of SAG core genes not found**	**Total SAG genes analyzed**	**% of SAG core genes found**
*S. gordonii*^a^	1073^*^	72	1145	93.71
*S. sanguinis*^b^	1069	76	1145	93.36
*S. mitis*^c^	992	153	1145	86.64
*S. pneumoniae*^d^	954	117	1071	89.08
*S. suis*^e^	969	148	1117	86.75
*S. agalactiae*^f^	956	163	1121	85.43
*S. pyogenes*^g^	887	209	1096	80.93
*S. mutans*^h^	958	180	1138	84.18

*This table was constructed using results from OrthoMCL; ^a^*S. gordonii* analysis included 1 strain; ^b^*S. sanguinis* analysis included 1 strain; ^c^*S. mitis* analysis included 1 strain; ^d^*S. pneumoniae* analysis included 12 strains; ^e^*S. suis* analysis included 6 strains; ^f^*S. agalactiae* analysis included 3 strains; ^g^*S. pyogenes* analysis included 13 strains; ^h^*S. mutans* analysis included 2 strains.

### Differences in protein functional classifications within SAG

**Figure 4:**
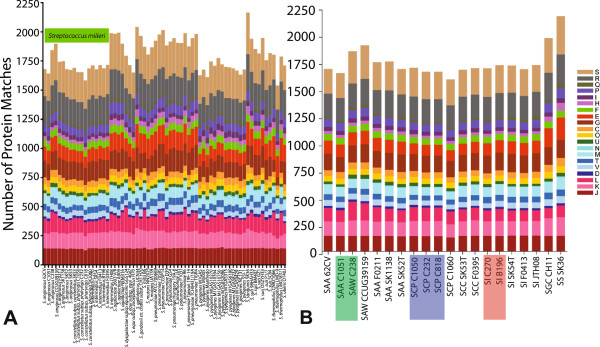
**Comparison SAG to the genus *Streptococcus *based on COG categories. A)** eggNOG comparison of available *Streptococcus* genomes from NCBI, SAG are highlighted with a green bar. **B)** Comparison of all SAG genomes (SAW; *S. anginosus* subsp. *whileyi*; SAA; *S. anginosus* subsp. *anginosus*; SCP, *S. constellatus* subsp. *pharyngis*; SCC, *S. constellatus* subsp. *constellatus*; SI, *S. intermedius*) with the most closely related *Streptococcus* strains (SS; *S. sanguinis*, and SGC; *S. gordonii*), strains sequenced in this study are highlighted with: red for SI, blue for SC, and green for SA. Only functional categories with at least one protein present are represented in the graphs. Significant BlastP hits were filtered using the following criteria: 80% PID over a minimum of 80% of the protein length or proteins over 100 amino acids in length with a minimum of 50% length and 30% PID to eggNOG categories. Category abbreviations as follows: J, Translation, ribosomal structure and biogenesis; K, Transcription; L Replication, recombination and repair; D, cell cycle control, cell division, chromosomal partitioning; V, Defense mechanisms; T, Signal transduction mechanisms; M, Cell wall/membrane/envelope biogenesis; N, Cell motility, U, Intracellular trafficking, secretion, and vesicular transport; O, Posttranslational modification, protein turnover, chaperones; C, Energy production and conversion, G, Carbohydrate transport and metabolism; E, Amino acid transport and metabolism, F Nucleotide transport and metabolism; H, Coenzyme transport and metabolism; I, Lipid transport and metabolism; P, Inorganic ion transport and metabolism; Q, Secondary metabolites biosynthesis, transport and catabolism; R, General function predicted only; S, Function unknown.

Protein functions for the ortholog groups (discussed above) were analyzed observing COG superfamily protein for all 66 *Streptococcus* reference strains this revealed that all strains had a similar distribution of proteins within the COG categories (Figure 4). Indeed previous studies have revealed the majority of *Streptococcus* strains have a similar percentage of their genome dedicated to each COG category [29-31]. Analysis of the total predicted coding ORFs shows that approximately 68% (66.5 to 70.6%) of all proteins in SAG code for a characterized protein from the COG superfamily of proteins, except for in SCP SK1060 (for reasons discussed above), which has only 51% of its proteins hitting to characterized proteins. Focusing on the most closely related strains to SAG reveals that *S. gordonii* and *S. sanguinis*, both have approximately 68% of their genomes coding for characterized COG superfamily proteins (Figure 4B).

Comparing SAG to *S. gordonii* and *S. sanguinis* there are only a few noticeable differences in the number of proteins within different COG categories (Figure 4B). Within SA there is an increase of replication, recombination, and repair proteins for SAW C238 (172) and SAW CCUG39159 (159), compared to an average of 117 for the other strains in Figure 4B. This difference is likely due to the increase in phage-related proteins within a recently identified SAW subspecies, which may have increased propensity for the uptake of foreign DNA (largest SAG genomes in this study) as compared to SAA (Figure 1A). There is a decrease in amino acid transport and metabolism proteins (Group E), and poorly characterized protein (Groups R and S) in SAG (112 and 472) compared to *S. gordonii* (173 and 570) and *S. sanguinis* (202 and 620). Some of these differences are due to the size of the genomes, as the genomes for both *S. gordonii* and *S. sanguinis* are larger than the SAG genomes, accounting for some of the increase in proteins found. In *S. mutans*, COG groups L, E, R and S are also known to be variable within the genus, with many proteins from these groups present within the accessory genome for *S. mutans*[46]. The analysis of these differences will provide insight into the species-specific characteristics for SAG.

### Orthologs identified as SAG-unique signatures

The delineation of SAG as a unique group of *Streptococcus* species relies on the presence of genomic material not present within other *Streptococcus* species. SAG-unique signatures were identified as regions conserved within SAG and absent in all other *Streptococcus* strains. A total of 688 ortholog groups were present in at least one strain of SAG and not in any of the other *Streptococcus* strains included in this study. Small conserved regions were identified from the overall large COG figure and a drill down figure focusing on the potential unique regions within SAG was created (Additional file 5). Of the 114-ortholog groups that were identified within SAG, only eight unique proteins were conserved within most of the sequenced SAG strains (Table 4). These eight proteins had <50 percent identity (PID) to anything in the NCBI protein database and <40 PID to any streptococcal protein (Table 4). This analysis was conducted for each individual species with SA, SI and SC having 1, 14 and 42 ortholog groups respectively that were unique to each species. A number of orthologs were unique to each strain of SAG, however, almost all of these genes encoded for hypothetical or conserved hypothetical proteins (Additional file 7). Very few unique proteins were observed for SA and SI*.* Interestingly, the unique genes for SC had a G + C content of 33.4%, which is lower than the average G + C content for sequenced SC strains (38.1%), suggesting these genes were likely acquired via horizontal transfer. A small number of unique genes found in SAG is not unexpected since a large number of *Streptococcus* strains have been sequenced to date; further analysis of these proteins may provide specific markers for detection or typing assays.

**Table 4:** Unique genes found in SAG strains, as determined by OrthoMCL analysis

**Locus name**^**a**^	**Length (AA)**^**b**^	**Gene product**	**% G + C**	**COG #**^**c**^	**% HSP**^**d**^	**PID**^**e**^	**Organism**^**f**^	**Accession #**
SCRE_0567	128	Conserved hypothetical protein	22.22	3514	108.5	37	*S. macacae*	ZP_09135394
SCRE_0686*	202	Putative phosphoglycerate mutase	36.95	4151	100.0	50	*Paenibacillus* sp	YP_003241041
SCRE_1286	126	Conserved hypothetical protein	32.55	4048	100.0	41	*Leuconostoc fallax*	ZP_08312902
SCRE_1287	278	Conserved hypothetical protein	38.47	4049	100.0	57	*Lactobacillus casei*	YP_805494
SCRE_1374*	316	Tagatose 1,6-diphosphate aldolase	39.43		100.0	65	*Pasterurella mutocida*	ZP_18466110
SCRE_1851*	333	Conserved hypothetical protein	38.82	3609	100.6	28	*S. gordonii*	YP_001449372
SCRE_1887*	229	Triose-phosphate isomerase	28.41	4057	100.0	40	*Leuconostoc citreum*	YP_001728668
SCRE_1888	179	Conserved hypothetical protein	27.96	3880	100.0	48	*Bacillus sp*	ZP_01723913

*Genes found in all sequenced SAG from GenBank and this study. ^a^*S. constellatus* subsp. *pharyngis* C232 was used as reference for all SAG strains; ^b^Length of protein within SAG strains in amino acids; ^c^COG number generated during orthoMCL analysis; ^d^Percent coverage of protein from SAG compared to best match in NCBI database using BlastP; ^e^PID is equal to the percent aa identity for the best match using BlastP from NCBI ; ^f^Organisms with best protein match to a non-SAG strain from NCBI database using BlastP.

### SAG virulence factor repertoire identified for future pathogenomics investigations

**Figure 5:**
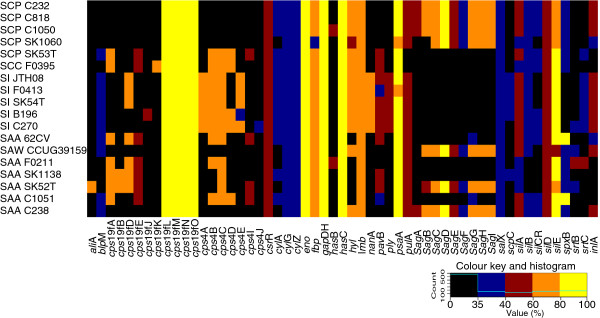
***Streptococcus *virulence protein detection within SAG, *S. gordonii *and *S. sanguinis *strains.** A total of 66 *Streptococcus* genomes (Additional file 2) were queried using a custom virulence database (VirDB) of 189 known *Streptococcus* virulence determinants (Additional file 6) and a heatmap was constructed to demonstrate relatedness of virulence determinants within each queried strain. The legend on the lower right hand corner depicts the PID of the ortholog from the specific strain to the reference protein within the VirDB and the sum of hits (histogram). The heatmap uses 5 colors to represent percent protein identity as follows: SCP, *S. constellatus* subsp. *pharyngis*; SCC, *S. constellatus* subsp. *constellatus*; SI, *S. intermedius*; SAA, *S. anginosus* subsp *anginosus*; SAW, *S. anginosus* subsp*. whileyi*; Black loci not present (0-35%), Blue (36-40%), Red (40-60%), Orange (60-80%) and Yellow (80–100).

Owing to a paucity of genome and experimental data, limited virulence traits have been previously identified for the SAG, and studies to date have focused on specific virulence traits and not their global virulence potential [3,22-25,27,47]. The curated genomic sequence data sets herein provide the opportunity for *in silico* ortholog gene searches to identify potential virulence traits within SAG. A custom virulence database (VirDB) constructed for this study contains 189 known or putative virulence factors from the genus *Streptococcus* (Additional file 8). VirDB was used to query all sequenced *Streptococcus* strains (Additional file 3) to identify virulence genes within the *Streptococcus* pan-genome (Figure 1). A more detailed analysis of the putative virulence genes within *S. gordonii* and *S. sanguinis* is also presented (Figure 5). There are 55 virulence proteins identified within SAG that had greater than 35 PID over at least 50% of the protein when compared to this virulence database (Additional files 9, 10 and 11). The importance of using these respective cut-offs was to limit the over-inference of short domains or incomplete proteins in our analysis. Overall, there were 16 known *Streptococcus* virulence proteins conserved within the sequenced SAG strains and each individual SAG strain contained 27 to 36 virulence proteins. The breakdown of virulence gene by SAG species was 30–34 (SA), 30–35 (SC) and 27–36 (SI). The overall division of virulence traits showed that SI and SC share greater than half of their virulence traits, although the overall repertoire of virulence traits differed between the two species. There was less conservation of virulence traits among the seven strains of SA*,* with some strains showing greater similarity to either SC or SI for their overall virulence gene composition (Figure 5). Although the phylogenetic groupings in this study are from our core-SNP method, the examination of the genes reveals the same similarities mentioned above.

Five of the 55 virulence proteins newly identified within SAG are inferred to be adhesion proteins including: a fibronectin binding protein (Fbp54), important in adhesion for *S. gordonii*[48], a *S. pneumoniae* and *S. mitis* surface adhesion protein (PsaA) [49], a laminin-binding protein important for adherence in GBS [50], pullulanase protein important in *Streptococcus* adhesion [51] and *Streptococcus* enolase a strong plasminogen-binding protein [52].

Twelve loci involved in invasion or evasion from host proteins were also identified in SAG. All five proteins encoded for by the *Streptococcus* invasion locus *sil*A to *sil*E [53], four capsule proteins (Cps19FL to O) from *S. pneumoniae*[54], hemolysin proteins from the *S. agalactiae* hemolysin loci *cyl*Z and *cyl*G [55], and UDP-glucose pyrophosphorylase protein (HasC) [56], were present in the conserved SAG virulence proteins. Finally, three regulator function proteins were present, including a two component response regulator CsrR (CovR), known to regulate expression of extracellular carbohydrates in *Streptococcus*[57]; a protein with homology to salivaricin-A (SalX), a bacteriocin known to inhibit growth of streptococci [58]; and GAPDH an immunomodulatory protein important in streptococcal colonization [59].

Some virulence proteins were present in more than one species of SAG revealing that the SAG virulence repertoire may vary with strain. Homologs for *S. pneumoniae* polysaccharide capsule operon (Cps4) [60]; four proteins from the hemolysin complex from *S. agalactiae* (Cyl) [55] and a *S. pneumoniae* surface-expressed adhesion protein (PavB) [61] were found in SI, SCC and some SA. Interestingly only some SA (C238, CCUG39159 and SK52T) and SCP contained homologs to *sag*A through *sag*I that form the streptolysin S cytolytic toxin complex of GAS. Streptolysin S is a strong cytolytic toxin that provides the ability for transepithelial migration [62] and *sag*A homologs have recently been shown to confer β-hemolytic activity in SA [3]. The cytolytic function can be detected via β-hemolysis on sheep’s blood agar plates. Strains predicted to be β-hemolytic based on genomic analysis (SAW C238 and SCP C818, C232 and C1050) were congruent with β-hemolytic phenotype observed on sheep blood (results not shown).

There was also a homolog present in SAG to a hyaluronidase precursor protein (HylA) that provides the ability to survive on host hyaluronic acid as a sole carbon source, as well as aiding in bacterial spreading by allowing for detachment from biofilms [27]. This gene was found in all SC and SI strains and SAW strains, and the presence of the HylA protein was confirmed through traditional phenotypic growth testing for in house sequenced SAG strains [9].

Internalin A (InlA) is a major invasion protein from *Listeria monocytogenes* that mediates the attachment and invasion of hepatocytes by *L. monocytogenes* and is encoded by the *inl*A gene [63]. A homolog to this gene has been found in *Streptococcus* spp. and was termed the streptococcal leucine-rich (Slr) protein [64]. A homolog to this protein was identified in all SCP, SI and some SA (C238, 62CV and CCUG39159), and is highly conserved within SAG having 97.4% nucleotide identity and 97.2 PID. Internalin A orthologs have also been identified in many other sequenced streptococcal species including *S. sanguinis* VMC66 [GenBank: EFX94353.1], *S. pyogenes* MGAS8232 [GenBank; AAL97968.1] and *S. agalactiae* 2603 V/R [GenBank; NC_004116]. Within sequenced *S. sanguinis*, *inl*A is found inserted between *pyr*R and a hypothetical protein, while in *S. pyogenes* this gene is located between *met*K and *bir*A and in *S. agalactiae* it is located between *lep*A and a histidine diad domain protein encoding gene. The region around the *inl*A gene is conserved in all SAG, with *inl*A inserted between *pyr*R and a conserved hypothetical protein as previously seen in *S. sanguinis*. In SAG without *inl*A, the *pyr*R and the gene encoding the conserved hypothetical protein are present, thus it appears that the *inl*A was lost or never integrated into some SA and SCC. In *S. gordonii* [GenBank: NC_009009], SA 62CV [GenBank: EFW07950.1] and SC SK1060 [GenBank; EGV09572.1], there are remnants of a leucine-rich protein that has been truncated located next to *pyr*R, which shows that *inl*A has been gained and lost in some *Streptococcus* strains. It has been shown in *L. monocytogenes* that loss or truncation of the *inl*A gene causes decreased invasive ability [65]. Similar results were shown for *slr*, where an isogenic GAS strain lacking *slr* was significantly less virulent in a mouse model and more susceptible to phagocytosis by human polymorphonuclear leukocytes [64].

Two virulence genes were identified in SI that were not found in any of the other SAG. Both genes have been previously identified including; *nan*A, a sialidase A precursor that may influence host bacterial interactions [47], a pneumolysin-like protein (Ply), identified as intermedilysin, which is a human erythrocyte specific cytotoxin [4,21]. There were no virulence traits found to be specific to either SC or SA. Indeed, the use of VirDB has identified numerous virulence genes within SAG that may allow SAG to adhere, invade and spread within the host.

### Evaluation of colonization potential: a genetic look at SAG LPXTG proteins

Bacterial attachment to host cells is essential in host colonization. Colonization occurs through interactions between bacterial surface-exposed proteins and host cell receptors [66]. One of the most common motifs found in bacterial cell surface-exposed proteins is the LPxTG motif. The motif anchors the C-terminus of these externally facing surface fibrillar proteins [67]. These LPxTG motif proteins are covalently attached to the cell wall by sortases, with sortase A (SrtA) being the most common within streptococci [68]. Many LPxTG motif proteins have been associated with virulence in Gram-positive microorganisms, however many also have no known associated function [20,68]. Each SAG strain had at least one *srt*A sortase ortholog, with all SI having two *srtC* orthologs. SA F0211 is the only SA strain to have multiple sortases, with a SrtA and two SrtC orthologs. Increased sortase function may increase virulence potential through assembly of surface structures [69].

A total of 58 LPxTG motif proteins were identified within SAG, however, only 50 of these proteins had a signal peptidase in addition to the LPxTG motif to allow expression on the cell surface. The number of LPxTG motif proteins in SAG strains was in the mid range for *Streptococcus* spp. ranging from 14 to 22 [28,29,32,70]. SA had the most LPxTG motif proteins with 21 and 22 for SAW C238 and C1051 respectively, while SC and SI ranged from 14 to 17 (Additional file 12). One LPxTG motif protein, hyaluronate lyase precursor protein (HylA) was found in all SI, SC and SAW strains. This protein is known to be an important virulence factor as discussed above. Twelve of the LPxTG motif proteins had collagen-binding domains, the same number found in *S. equi* subsp. *zooepidemicus*[32], and thus could be involved in SAG virulence. Also identified were eight proteins important as either pili or in fibrinogen-binding. The presence of numerous potential adherence proteins is common for oral microbiota, and thus, high numbers of these proteins within SAG is not unexpected.

### Genetic analysis of two component (histidine kinases/response regulator) systems (TCS)

Bacterial survival is dependent on a pathogen’s capacity to sense and respond to their environment. Two-component histidine kinase systems (TCS) have been shown to play vital roles in virulence as well as the ability to respond to specific environmental signals [71,72]. In total there were 14 TCS found in SAG, 9 of which were conserved in all SAG strains, although the response regulator (RR) for SAGTCS6 is truncated in SAW C238 and the SAGTCS6 histidine kinase (HK) is truncated in SCP strains. *S. intermedius* and SAW had the most TCS with 13, while SCP had the least with 10 (Table 5). These numbers are similar to other sequenced *Streptococcus* strains including: 14 in *S. sanguinis*[31], 13 in *S. pneumoniae*[73-75], 14 in *S. mutans*[28,76], 13 in *S. pyogenes*[29] and 17 in *S. agalactiae*[70].

**Table 5:** **Comparative summary of two component system (TCS) histidine kinases identified in SAG with other *Streptococcus *species**

**SAGTCS**^**1**^	**Equivalent TCS HK in *S. pneumoniae***^**2**^	**Gene name**	**Linked to virulence**	**SAG strain found in**	**Loci**^**3**^	**Best non-SAG BlastP results**	**PID**^**4**^	**Accession #**
1	9	zmpSR	Yes	All SAG	SIR_0800	*S. sanguinis*	69	YP_001035081.1
2	5	*cia*H	Yes	All SAG	SIR_0858	*S. oralis*	70	EGL88510.1
3	2	*vic*K	Yes	All SAG	SIR_0929	*S. cristatus*	84	ZP_08058686.1
4	None		ND^a^	All SAG	SIR_1092	*S. sanguinis*	67	EGG40097.1
5	13	*blp*H	Yes	All SAG except SA^b^ SK1138, SA F0211 and SA SK52T	SIR_1152	*S. equi*	50	YP_002746951.1
6	1		Yes	All SAG	SIR_1196	*S. cristatus*	63	ZP_08059673.1
7	3		No	All SAG	SIR_1292	*S. cristatus*	67	ZP_08060583.1
8	None		ND	All SAG	SIR_1635	*S. cristatus*	89	ZP_07864009.1
9	8		Yes	All SAG	SIR_1771	*S. mitis*	64	ZP_07642266.1
10	12	*com*D	Yes	All SAG	SIR_1900	*S. cristatus*	59	ZP_08060066.1
11	6		Yes	SI^c^ and SA	SIR_0289	*S. sanguinis*	65	EGJ40992.1
12	7		Yes	SI and SA C1051	SIR_1062	*S. cristatus*	71	ZP_08060320.1
13	11		No	SI and SCC^d^	SIR_1373	*S. sanguinis*	96	EGF13754.1
14	None		ND	SA C1051, SA 62CV and SA SK52T	SAIN_0922	*S. oralis*	95	ZP_13522336.1

^1^Two component system histidine kinases systems found in SAG; ^2^The equivalent TCS found in S. pneumoniae as reviewed by Paterson et al. [74] , SAG orthologs found using blast hits from NCBI; ^3^All SAGTCS present in more than one SAG are listed using the loci from histidine kinase from SI B196 since this strain had the most TCS orthologs.; ^4^PID is equal to the percent aa identity for the best match using BlastP from NCBI ; ^a^ND; No data for this HK, ^b^SA = *S. anginosus*, ^c^SI = *S. intermedius*, ^d^SCC = *S. constellatus* subsp. *constellatus*.

Thirteen of the 14 TCS found in SAG were found to have orthologs in other species of *Streptococcus* including nine orthologs to TCS that have been linked to virulence in *S. pneumoniae*[75]. Of these nine, four are well characterized, including VicRK, CiaRH, ComDE, and BlpR (Table 5). These TCS are important to *S. pneumoniae* virulence, quorum sensing, competence, bacteriocin production, and stress response [71,77]. Four additional SAG TCS (designated herein as SAGTCS1, SAGTCS6, SAGTCS9, and SAGTCS11) show homology to less characterized TCS that are also important for virulence in S. pneumoniae [78,79]. Another TCS sequence (SAGTCS13) is poorly characterized showing the most similarity to a characterized TCS from *S. mutans*[76]. SAGTCS13 has been disrupted in SCP and SA except SK52T, CCUG39159 and C1051, to leave only a truncated portion of the HK with an absent cognate RR. This truncation appears to be due to the insertion of the SAG operon within these strains of SAG, as a complete HK/RR system is observed in all strains that lack the SAG operon; the importance of this TCS has not been well characterized [74]. Finally SAGTCS14 was only found in SA C1051, 62CV and SK52T, with homology to uncharacterized TCS from *S. oralis*, *S. mutans*, *S. macacae* and *Lactobacillus salivarius*. The importance of TCS in many aspects of virulence is well known and shows the virulence potential for SAG, however, more work is required to fully characterize TCS within SAG.

### Potential natural immunity conveyed by clusters of regularly interspaced short palindromic repeats (CRISPRs) within SAG

Clusters of regularly interspaced short palindromic repeats (CRISPRs) contribute to bacterial immunity to invasion from foreign DNA such as bacteriophage and plasmid DNA [80]. CRISPR analysis in SAG strains showed that seven of the 18 strains analyzed contained CRISPRs (Additional file 13). Three of the seven strains had more than one CRISPR for a total of 12 CRISPRs identified within SAG (Additional file 13). Based on the Cas1 protein all CRISPRs except for one that had a Cas1 protein were of the CRISPR subtype II-A also known as the NMENI subtype [80,81].

**Figure 6:**
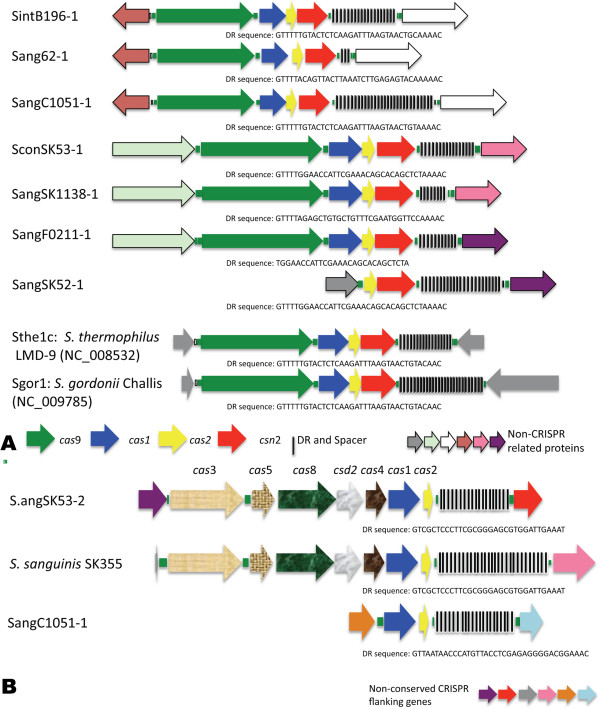
**Comparative analysis of the Type II-A (Nmeni) SAG CRISPR. A)** Schematic view of the Type II-A CRISPR regions identified within SAG. **B)** Schematic view of the Type I-C CRISPR region identified within SA. Similar colored arrows indicate gene loci conserved between SAG. The number of DR and spacer regions are indicated by black bars, with the number of bars correlating to number of regions present within each CRISPR.

The CRISPRs SintB196-1, Sang62-1 and Sang1051-2 were closely related to each other based on Cas1 AA identity and are all inserted between a transcriptional regulator (SI B196 SIR_0753, SAA C1051 SAIN_0897 and 62CV_1302) and a conserved hypothetical protein (LPXTG motif protein: SI B196 SIR_0758, C1051 SAIN_0902 and 62CV_1297). CRISPRs SconSK53-1, SangSK52-1, SangSK1138-1 and F0211-1 and F0211-3 were also similar to each other based on Cas1 AA identity. F0211-1 and F0211-3 may be part of the same CRISPR as inferred from their location within the genome; however, as this strain is only publically available as a draft WGS project data set, this inference cannot be confirmed. Gene composition was the same for all three CRISPR elements including known CRISPR associated proteins *cas*9 (*csn*1), *cas*1, *cas*2 and *csn*2 followed by the CRISPR direct repeat (DR) and spacer regions (Figure 6A). This genetic arrangement and Blast results suggests that this CRISPR is most closely related to the CRISPR subtype II-A family. CRISPRs with similar genetic content have been identified within *S. thermophilus*, *S. gordonii*, *S. suis* and *S. infantarius*[82]. The DR length was 28 to 36 bp for all three strains (it appears the smaller direct repeats are truncated versions of the larger direct repeats), while there was variation in the number of spacers present within each CRISPR (Additional file 13). The DR regions were similar for all subtype II-A CRISPR elements within SAG with the last repeat showing variation as has been shown for other CRISPR elements [80].

The CRISPR SconSK53-2 was the only non Type II-A CRISPR (identified as Type 1-C), which is located next to an integrase and is composed of *cas*3, *cas*5, *cas*8, *cas*7, *cas*4, *cas*1 and *cas*2 genes from 5′ to 3′ and followed by a CRISPR region with a DR of 32 bp and 19 spacers (Figure 6B). This type of CRISPR has been previously identified within *Streptococcus*[83], and showed the most similarity to CRISPR-associated loci from *S. sanguinis*, *S. mutans*, *S. parasanguinis*, and *S. pyogenes*. The localization next to an integrase gene and high similarity (85 to 93% AA identity) to a similar region within *S. mutans* LJ23 (GenBank NC_017768), while the surrounding area shows less similarity (<78% AA identity) suggests that this region was horizontally transferred into SCC SK53T.

*S. anginosus* C1051 had a total of three CRISPR regions, with Sang1051-1 and Sang1051-3 both appearing to be degenerate. Sang1051-1 has *cas*1 and *cas*2 genes and the CRISPR region with 21 DR of 37 bases in length (Figure 6B), similar to Sthe2c from *S. thermophilus* LMD-9 [82], however, compared to other Cas1 proteins there is less than 65% AA identity as compared to all other Cas1 proteins that have matches greater than 90% based on AA identity. The third CRISPR region, Sang1051-3, had three spacers of 28 bases (Additional file 13). Although neither CRISPR-1 nor CRISPR-3 had a full complement of CRISPR-associated proteins, the fact the CRISPR-1 had 21 spacers suggests that this CRISPR may still be functional [84]. *S. anginosus* C1051 and SI B196 did not have any detectable prophage integrated into their genomes. This may be due to the presence of multiple CRISPR elements within these genomes.

For the 12 CRISPRs described above, there were a total of 201 spacers (Additional file 14). Of these 201 a total of 50 had greater than 80% identity over 50% of their sequence to a sequence in GenBank. However, none of the spacers from SAG showed 100% DNA identity to anything in the NCBI nucleotide database, which is significant as it has been shown that 100% match is required for immunity to foreign DNA [85]. A total of 11 spacers showed >90% nucleotide similarity to previously identified Streptococcal-specific phages including five phages from *S. pneumoniae*; 11865, 8140, V22 [86], Cp-1 [87] and EJ-1 [88]; two phages from *S. pyogenes*, *phi*370.2 [34], and *phi*NIH1 [89]; streptococcus phage C1 isolated from Group C *Streptococcus*[90]; *S. oralis* phage PH10 [91]; *S. gordonii* phage PH15 [92]; and a phage from S. *gallolyticus* subsp. *gallolyticus* UCN34 [93]; (Additional file 14). All spacers were unique except for 16 spacers from SA F0211, this one spacer was located within each of the three CRISPRs identified for SA F0211, these spacers were similar to an integrative conjugative element (ICE) from *Vibrio fluvialis* Ind1 (ICEVflInd1) and showed similarity to genes encoding for pili and conjugative machinery. It appears that F0211 had multiple encounters with one or multiple conjugative elements similar to ICEVflInd1 and has acquired a means to prevent integration of this type of ICE. Perhaps CRISPRs have played a role in the evolution of SAG and may be responsible for the lack of foreign genetic material seen in some in-house sequenced SAG strains. We are currently investigating whether SAG CRISPRs might be variable enough to be useful in a subtyping scheme.

### Extensive horizontal gene transfer and accessory gene content in SAG

None of the SAG strains sequenced in this study possessed extracellular plasmids, which would indicate that this is not a common method of HGT among SAG strains; although loss of plasmid on in vitro culture is not uncommon and cannot be completely ruled out. This was shown in a previous study where only two plasmids were identified within 27 strains of SAG [5]. However, all strains from this study showed evidence of HGT via presence of prophage, insertion sequences, transposons and ICEs and those account for 12 to 28.5% of the total predicted coding sequences within the seven genomes sequenced for this study (Table 6; Figure 1; Additional file 2). The prophage and ICEs identified within this study are highlighted in Figure 1B-D and can be seen in the MAUVE analysis also (Additional file 2). These SAG mobile elements will be described in detail elsewhere (Olson et al., manuscript in preparation).

**Table 6:** SAG accessory regions

**Genome**	**Genome size (nt)**	**# AR**^**1**^	**Combined size ARs (nt)**	**AR% of Genome**	**AR CDS**	**HGT ARs**^**2**^	**HGT CDS**^**3**^	**Combined Size HGT ARs (nt)**	**% of Genome**
SA C1051	1911706	47	437231	22.87	353	18	218	306042	16.01
SAW C238	2233640	53	745460	33.37	608	26	481	636082	28.48
SCP C232	1935414	34	476022	24.60	412	12	287	330294	17.07
SCP C818	1935662	34	476022	24.59	412	12	287	330294	17.07
SCP C1050	1991156	35	531952	26.72	461	14	344	402267	20.21
SI C270	1960728	47	524216	26.74	437	7	202	243715	12.43
SI B196	1996214	52	554383	27.77	470	11	234	279114	13.98

^1^AR; Accessory region, ^2^HGT AR, Accessory region with evidence of horizontal gene transfer; ^3^HGT CDS, total number of predicted coding regions within the HGT ARs. SA, *S. anginosus* subspecies anginosus; SAW, *S. anginosus* subspecies whileyi; SCP *S. constellatus* subspecies pharyngies, SI; *S. intermedius*.

### Investigation of SAG natural competence

Uptake of naked DNA from the environment, known as natural competence, allows bacteria to survive and thrive in variable environmental conditions. Natural competence has been studied in-depth for the streptococci with *S. pneumoniae* serving as the model organism [94]. The complexity of natural competence systems and diversity of streptococcal competence genes has made *in-silico* prediction of natural competence difficult [95]. Genome transcriptomics and tiling microarrays have shown that there are 22 essential genes required for natural transformation in *S. pneumoniae*[96]. Orthologs to 21 of the essential competence genes were identified in all seven in-house sequenced SAG (Additional file 15). For all remaining WGS strains, there were truncated or absent genes likely owing to sequence errors; however, this would have to be experimentally confirmed. The only homolog absent in all strains was *com*W, which is also missing in the naturally competent *S. zooepidemicus*[97], *S. sanguinis*[31] and *S. mutans*[98] suggesting that it may not be absolutely required for natural competence.

All SCP examined to date (*n* = 34) are not naturally competent, but competence has been shown in SI (Lacroix, Grinwis and Surette, unpublished data). Sequence analysis has revealed that the lack of natural competence in SC may be due to an insertion within *com*EA gene. This truncation results in a gene product approximately half that of the original *com*EA and when this gene is inactivated in *S. pneumoniae*, competence is abrogated [96]. The detected insertion has been experimentally validated using Sanger sequencing. All SCP strains had a nine nt in-frame deletion in the *com*D gene. This has been found to be a feature of all SCP (*n* = 34), but does produce a functional ComD (Lacroix, Grinwis and Surette, unpublished data). The only other difference seen in the complement of competence genes within SAG was the number of *com*X orthologs. For most *Streptococcus* strains there are two *com*X orthologs (*com*X1 and *com*X2), for the SAG there were three *com*X loci in most strains. However, one copy of *com*X was truncated in SCP C1050, and in some of the publicaly available WGS strains included in our analysis (Additional file 16). Further functional studies will have to be completed in SAG to identify and characterize which competence proteins are essential.

### Microevolution within SAG

Two strains (SCP C232 and C818) that were sequenced within this study were cultured from the same individual almost one year apart. These isolates were both the numerically dominant strain present during a pulmonary exacerbation. Genomic comparisons revealed that these strains were almost identical, differing by only 18 SNPs (Additional file 17). The SNPs were found within intergenic regions (6), and causing both synonymous (3) and non-synonymous mutations (9). Two of the non-synonymous mutations introduced stop codons, resulting in two truncated genes within SCP C818; one is a putative ABC transporter (ATPase portion) while the other is a 3-dehydroquinate dehydratase (*aro*D). Mutations in *aro*D have been extensively characterized in *Salmonella typhi*[99] and have been shown to render bacteria auxotrophic for aromatic amino acids p-aminobenzoate (pABA) and 2, 3-dihydroxybenzoate. This results in the inability to produce ubiqinone and menaquinone causing cellular respiration defects [100], as well as defects in the cell envelope [101]. Indeed, it has been shown that aromatic amino acids are abundant within the sputum produced within the lungs of CF lung patients and thus may provide an adequate source of these essential nutrients [102]. Further studies will be required to determine the importance of this *aro*D mutation within SCP C818. Two SNPs were located within a region of the *rpo*B gene known to cause rifampicin resistance [103]. Finally, a second type of divergence between SCP C232 and C818 were tandem repeat and microsatellite regions, which are potential targets for multilocus variable number tandem repeat analysis (MLVA). There were a total of five regions of increased/decreased copies of tandem repeats ranging in size from four to 41 nt (Additional file 18). Of these five regions, three are located in truncated genes, whereas the other two were within non-coding regions. Further analysis into potential MLVA targets within SAG is warranted. Although both these strains are highly similar, appreciating the evolution that occurred during a year in a CF lung is critical to understanding the fitness advantage required for chronic bacterial pathogens.

## Conclusions

This study presented the analysis and comparison of the whole-genome sequences for the three species within SAG, important pathogens with the capacity to cause serious infections throughout the body. Sequencing strains from both respiratory and invasive infections, we identified no clear differences in gene content between these types of isolates, suggesting that it may be host factors that promote certain types of infection as opposed to bacteria-specific virulence factors. There were only eight genes detected that were uniquely common to all seven in-house sequenced SAG; these were also found in most SAG draft genomes that are currently publically available. The comparison of SC, SI and SA strains revealed significant differences with respect to virulence factors, surface proteins and carriage of horizontal genetic elements, with SA showing the most intra-species variability. Horizontal gene transfer between SAG and other pathogens within their environment has clearly played a significant role in the evolution of species within SAG, which will need to be studied in far greater detail. The detailed comparison of microevolution within SAG has identified potential targets for molecular typing methods as well as potential research questions regarding survival of a bacterial pathogen within the lung of a CF patient. The generation of our high quality finished reference genomes for the seven in-house sequenced SAG strains will provide a valuable resource for the analysis of future SAG draft genomes. This comparative genomic analysis provides a key genetic framework for assessing and understanding the molecular events contributing to SAG pathogenesis.

## Methods

### Bacterial isolates

Four respiratory SAG isolates were cultivated on McKay agar from adult patients at the time of a pulmonary exacerbation. The remaining three isolates were from infections at other body sites to provide a comparison between respiratory and non-respiratory (invasive) SAG strains. A total of seven SAG strains were sequenced with at least one respiratory and invasive strain from each of the three species within the SAG. Three SCP strains were sequenced: C232 and C818, both respiratory strains isolated from one individual almost a year apart, and C1050, an invasive isolate from a non-related individual. Two SI strains were sequenced: C270, a respiratory isolate, and B196, an invasive isolate obtained from a CF patient coinciding with a pulmonary exacerbation. Finally, two SA strains were sequenced: SAW C238, a respiratory isolate, and SAA C1051, an invasive isolate (Table 1). All isolates were obtained in accordance with the University of Calgary ethics approval and written approval was obtained for participation in the study from all human subjects providing bacterial isolates.

### Chromosomal DNA isolation

Strains were cultured on Columbia blood agar plates (CBAB) or Brain heart infusion (BHI) agar and incubated for 24 hr at 37°C plus 5% CO_2_. A single colony was inoculated into 20 mL of BHI broth and incubated as above. These cultures were then centrifuged at 5000 × g for 30 min and washed three times with sterile PBS. Cells were re-suspended in 1 mL of sterile PBS and lysed by physical disruption using the MiniBeadbeater-8™ (BioSpec Products, Inc). DNA was purified from lysates using standard phenol: chloroform and ethanol precipitation. All genomic preps were run on an agarose gel to ensure chromosomal integrity. Finally, the DNA was quantified using the Qubit® (Invitrogen, Burlington, ON).

### Genome sequencing, assembly, and Gap closure

Genomic DNA was sequenced using the Roche GS20 standard platform as per manufacturer’s protocols (454 Life-sciences, Brandford, CT). For gap closure, fosmid libraries were created as per manufacturer’s protocols using the CopyControl™ Fosmid Library production kit (Epicentre Technologies, Madison, WI) and Sanger sequencing was done on selected clones. Traditional PCR was done using proof-reading (HiFi platinum *Taq*; Invitrogen*) Taq* polymerase as per manufacturer’s protocols. PCR products were purified using the QIAquick PCR purification kit (Qiagen, Mississauga, ON) and sequenced with an ABI3730XL capillary electrophoresis instrument (Applied Biosystems, Foster City, CA). After sequencing on the Roche GS20 genome sequencer the raw reads were assembled using the Newbler assembler software package v1.0.53.17. After closure of the genome to a single contigous sequence, the *ori* was located by performing a simple Blastp using *dna*A as a reference.

### Sequence errors and SNP confirmation

Potential sequence errors attributed to homonucleotide runs were manually tested in SCP C232 using primers designed to flank the region of interest. PCR amplification was conducted using Invitrogen HiFi Platinum proof-reading *Taq* polymerase (Invitrogen), following manufacturer’s instructions, with 1 μM of each oligonucleotide and the following thermocycling conditions: 94°C for 5 min, 40 cycles of 94°C for 30 s, 55°C for 30 s and extension at 68°C for at least 30 s (time varied for larger size fragments) followed by 68°C for 5 min. All SNPs found between SCP C232 and C818 were confirmed by Sanger sequencing as described above. For all other SAG strains (except SCP C232), a combination of standard sequencing and Illumina sequence technology were applied to correct base calling errors caused by homonucleotide runs. Illumina sequencing runs were completed at the Iowa State University DNA facility using an Illumina GAII, (Illumina Inc, San Diego, CA). For Illumina sequencing, 5 μg of genomic DNA was submitted. The DNA facility created libraries and bar-coded five strains (SCP C1050 and C818, SAA C1051 and SI C270 and B196) which were then pooled in a single lane using 36 bp reads, while SAW C238 was run in a single lane using 75 bp reads. To ensure that Illumina data was reliable, 100 regions showing variation between the GS20 data and Illumina data were analyzed by Sanger sequenceing. In all cases, the Illumina data was observed to be correct. Illumina data and GS20 data were aligned and co-analyzed using CLC Genomics Workbench version 4.0 (CLC genomics, Cambridge, MA).

### Genome auto-annotation

Draft and finished genomes were automatically annotated using an in-house version of the GenDB 2.2 genome annotation system. In the annotation pipeline, genes are predicted using a combination of CRITICA [104] and Glimmer 3.0 [105]*de novo* gene predictors. Locations of Ribosomal Binding Sites (RBS) were also predicted via CRITICA. RNAmmer [106] and tRNAScan-SE [107] were used to predict all rRNA (5S, 16S, 23S) subunits and tRNA, respectively. Functional observations were collected from BLASTN [108] alignments to the NCBI nucleotide (nt) database [109], and from BLASTP [108] alignments to the Kyoto Encyclopedia of Genes and Genomes (KEGG) [110] and the NCBI non-redundant protein (nr) database [109]. Observations related to protein families were collected using PSI-BLAST [108] alignments to SWISS-PROT (sp) [111] and Clusters of Orthologous Groups (COG) databases [112]. Conserved protein domain observations were collected as RPS-Blast [108] alignments to the Conserved Domain Database (CDD) [113]. Additional observations collected for each CDS included Hidden Markov Model protein sequence classification searches against the TIGRFAM [114] and PFAM [115] databases, membrane spanning helices searches via EMBOSS helix-turn-helix [116] TMHMM [117] and presence of signal peptide sequences [118]. All functional observations were analyzed within the annotation system using a set of pre-defined heuristics to automatically assign a gene name and biological role for each CDS, when possible. Bi-directional BLASTP [108] for all predicted CDS was run between each pair of genomes and within each genome to identify potential orthologs and paralogs, respectively.

All intergenic regions (with a 25 base pair (bp) elongation on either end) were analyzed through a separate customized pipeline for identification of potential reading frame shifts, short genes overlooked by the automatic pipeline, or genes in regions of localized atypical nucleotide composition. Regions with BLASTX [108] alignments and EMBOSS getORF [116] open reading frames (ORF) were identified as potential CDS regions, run through the function prediction pipeline and automatically marked for manual curation.

### Manual curation of SAG genomes

All predicted CDSs were manually inspected with their GenDB observations to validate inference of the auto annotation. Predicted genes were inspected for potential frameshift errors and alternative start sites. All potential frameshift errors were experimentally validated with Sanger sequencing (described above). Orthologs between genomes were multi-annotated manually with inference from a chosen reference genome using the GenDB ortholog finding tool based on ClustalW multiple alignments of sequences flagged from the Bidirectional Blastp observations. To facilitate annotating the genomes in a timely manner, sequencing corrections and annotation were finished concurrently. The final version of each corrected sequence was re-mapped to the annotations and observations using custom perl scripts built to work within the GenDB system.

### Accession numbers

The manually curated, high quality finished genomes sequenced generated within this study have been deposited at GenBank with the accessions, SAA C1051 [GenBank: CP003860], SAW C238 [GenBank: CP003861], SCP C232 [GenBank: CP003800], SCP C818 [GenBank: CP003840], SCP C1050 [GenBank: CP003859], SI B196 [GenBank: CP003857], SI C270 [GenBank: CP003858].

Genome visualization and analysis

The pan-genome analysis was done using Gview version 1.6 [119] with SAW C238 as the seed genome and adding all SAG genomes as listed in Figure 2 to create a pangenome reference, this pangenome was then used to compare all individual SAG genomes to the pangenome using a BLAST atlas with the default settings on Gview version 1.6 [119]. Blast atlas for each of the species within SAG were constructed by using the basic setting on Gview version 1.6 and creating a circular comparison with SAW C238, SCP C232 and SIB196 as the reference for SA, SC, and SI respectively. MAUVE version 3.2.1 [120] was used with default settings to construct linear views of the SAG chromosomes.

### Phylogenetic analysis

Orthologs were identified by OrthoMCL v2.0.2 [40]; for the in-house core-SNP pipeline those orthologs present as single copies and common to all data sets were included for analysis. Each orthologous group was aligned using ClustalW (v1.8.2) and manually edited to correct for incorrectly predicted start sites. A subset of SNP loci present among all data sets (core) were identified and used to generate a meta-alignment using an in-house Perl script for downstream analysis. Alternatively a set of core gene alignments was generated using AMPHORA [37]. Of the 31 gene sets generated, 3 core genes (*rplB*, *rplD*, *rplL*) were discarded from further analysis owing to the fact they were not all present in all WGS genomes undergoing analysis. Phylogenetic trees were generated for comparison of each analysis method; in-house core-SNP pipeline, AMPHORA, 16S rRNA, *groEL*, and *rpoB* using Phylogenetic estimation using Maximum Likelihood (PhyML 3.0) [121] with nucleotide substitution models LG. To assess the stability of the tree branching patterns in *rpo*B, *cpn*60 and 16S rRNA gene trees bootstrap analysis with 100 pseudoreplicates was performed using evolutionary models and tree building as described above. The in-house core-SNP tree was analyzed using the approximate likelihood ratio test, using a selection threshold of 0.8, which is comparable to bootstrap supports of 75% [121]. The ratio of mean non-synonymous (dN) to synonymous substitutions (dS) per site (dN : dS ratio) within the two selected genes (*rpo*B,*cpn*60) was calculated using MEGA software using the Nei-Gojobori Method with Jukes Cantor correction [122,123].

### Ortholog analysis

OrthoMCL [40] was used to identify orthologous gene groups from the proteomes of 66 sequenced *Streptococcus* strains (Additional file 2). Sequences and annotations for the WGS projects were obtained from the Broad Institute download site (http://broadinstitute.org/annotation/genome/Streptococcus_group/GenomesIndex.html) and all other fully annotated *Streptococcus* spp. were obtained from NCBI [109], excluding the seven in-house SAG. A BLASTP [108] e-value of 1e 10^-5^ and percent match length of 50% were used as cut-offs in orthoMCL [40] analysis. Signature genes for each strain were appended to the orthoMCL output, since it does not report signature genes, defined as genes that are only present in one strain. VENN diagrams for ortholog groups were generated using an in-house Perl script. VENN diagrams do not represent all genes from all genomes due to the fact that accessory genes are not included.

### Clustered COG analysis

A comparative COG analysis was performed using a custom set of Perl scripts. One representative from each accessory gene group from the orthoMCL [40] output, as well as any non-orthologous predicted genes from each of the 66 *Streptococcus* genomes above, were blasted against the non-supervised Orthologous Groups (eggNOG) database [124]. Significant BlastP [108] matches were filtered using the following criteria: 80% PID over a minimum of 80% of the protein length or proteins over 100 amino acids in length, with a minimum of 75% length and 30% PID. Matches were scored on a binary scale, wherein a significant match score was assigned a value of one; non-significant matches were assigned a zero. Accessory gene matches to each COG group were hierarchically column-clustered with distance correlation. The resulting dendrogram was represented as a heatmap generated in R (http://www.r-project.org) using heatmap.2 from gplots package (http://cran.r-project.org/web/packages/gplots/index.html) with each tile shade representing a functional COG category associated with the gene group. Gene shading of black indicates exclusion from the gene cluster. The row dendrogram of the heatmap was not hierarchically clustered and simply represents the order of the phylogenetic tree resulting from the in-house core-SNP analysis (described above). Gene groups with no significant hits or hits to proteins with no assigned functional category from the eggNOG database [124] were excluded from the heatmap representation depicted.

### COG table

A list of the number of protein matches to the eggNOG database [124] matching a COG functional category for each of the 66 *Streptococcus* reference strains was generated. Significant BlastP hits were filtered using the following criteria: 80% PID over a minimum of 80% of the protein length, or proteins over 100 amino acids in length with a minimum of 50% length and 30% PID.

### CRISPR identification

Genomes were scanned for presence of CRISPRs using the online program CRISPRFinder and CRISPRcompar [125,126]. CRISPRs were classified based on their composition of CRISPR associated proteins [81]. Spacers were identified within the CRISPR regions and BlastN was used to determine if there were matches to known mobile elements within the GenBank database.

### Virulence analysis (Heatmaps)

A virulence database (VirDB) was constructed in-house using a combination of a literature search from public NCBI protein database [109] and *Streptococcus* specific virulence genes from the Virulence Factors of the Pathogenic Bacteria Database (VFDB), http://www.mgc.ac.cn/VFs/main.htm[127]. The VirDB was made non-redundant using CD-HIT [128] with default values, and then curated manually to ensure that genes from the same operon were not collapsed into a single entry. The initial virulence database was comprised of 234 genes, but later reduced to 189 virulence-associated genes. A protein BLAST [108] was performed using cut-off scores of 35% percent identity (PID) and highest scoring pair (hsp) length of 50% for the longest genes. A similar database was constructed to specifically identify TCS and LPxTG proteins within SAG. Heatmaps were generated in R (http://www.r-project.org) using a modified version of heatmap.2 as described above. Genome order for presentation of data was predefined by the in-house core-SNP pipeline (described above). Gene order was organized alphabetically, although genes within an operon were clustered together. Virulence genes that did not have hits to any genomes were eliminated from heatmaps in Figure 5 showing SAG, *S. sanguinis* and S. *gordonii*.

## Competing interests

The authors declare that they have no competing interests.

## Authors’ contributions

ABO performed manual annotation, genomic data analysis and wrote the manuscript. HK provided bioinformatics analysis, manual annotation, and contributed to writing the manuscript. CDS participated in study design, coordination, manual annotations of genomes, and provided critical analysis of manuscript. MEG participated in manual annotation, provided critical insights to data analysis and contributed to drafting of the manuscript. PM provided bioinformatics analysis for virulence analysis, VENN diagram analysis, overall support of bioinformatics tools, and aided in drafting the manuscript. CO and ST were instrumental in genome closure. MG provided input on design of project and contributed to analysis of data. SDT provided support for acquisition of genomic sequence data, genome closure, and overall project design. GV provided overall bioinformatics support. CRC and MGS conceived of the study, participated in design and coordination of the study. All authors read and approved the final manuscript.

## Supplementary Material

Additional file 1: Table S1 Genome coverage by sequence method for seven in-house sequenced SAG strains. Click here for file

Additional file 2: Figure S1 MAUVE analysis of SAG genomes. A multiple alignment of SA, SI and SC to highlight accessory regions and potential synteny within the genomes. MAUVE 2.3.1 was used with default settings with and a reference genome for each species; SAW C238 (SA), SCC C232 (SC) and SI B196 (SI). Click here for file

Additional file 3: Table S2 Genome summary of 66 *Streptococcus* strains used for comparative genomic analysis studies. Click here for file

Additional file 4: Figure S2 Genomic content analysis comparison of SAG to other clinically important species within the genus *Streptococcus*. A) *S. suis*, B) *S. pyogenes*, C) *S. agalactiae*, D) *S. pneumoniae*, E) *S. mutans* F) *S. mitis*, G) *S. sanguinis*, H) *S. gordonii*. Numbers within circles correspond to number of CDSs that are conserved within the strains analyzed or that are unique to each strain analyzed. Click here for file

Additional file 5: Figure S3 Clusters of orthologous group (COG) analysis of 66 *Streptococcus* genomes. The phylogenetic tree was constructed using an in-house core-SNP pipeline as described previously. This Figure is a small portion of the overall comparison of all orthologs representing COGs present or absent in SAG while absent or present within the majority of other analysed *Streptococcus*. The COG map was created using OrthoMCL, COG functional categories are shown on the far left portion of the Figure. The *Streptococcus* species used for analysis are listed in Additional file 2, Table S2. Click here for file

Additional file 6: Table S3 Comparison of gene content of SAG species to *S. gordonii* str. Challis substr. CH1 and *S. sanguinis* SK36. Click here for file

Additional file 7: Table S4 Genes unique to sequenced SC, SI or SA. Click here for file

Additional file 8: Table S5 Virulence genes used in virulence database to query SAG for potential virulence genes. Click here for file

Additional file 9: Table S6 *Streptococcus* constellatus genes with a match to the virulence gene database. Click here for file

Additional file 10: Table S7 *S. intermedius* genes with a match to the virulence gene database. Click here for file

Additional file 11: Table S8 *S. anginosus* genes with a match to the virulence gene database. Click here for file

Additional file 12: Table S9 SAG LPxTG proteins. Click here for file

Additional file 13: Table S10 Summary of CRISPRs found in SAG. Click here for file

Additional file 14: Table S11 Spacer regions within SAG CRISPRs. Click here for file

Additional file 15: Table S12 Comparative analysis of essential competence genes from *S. pneumoniae* TIGR4. Click here for file

Additional file 16: Table S13 Comparison of competence protein carriage within SAG. Click here for file

Additional file 17: Table S14 SNPs found within chromosomal sequence for SCP C232 as compared to SCP C818. Click here for file

Additional file 18: Table S15 Tandem-repeats and microsatellite differences identified between SCP C232 and SCP C818. Click here for file
