# Supplementary material for: Phylogenetic relationship and virulence inference of Streptococcus Anginosus Group: curated annotation and whole-genome comparative analysis support distinct species designation
Source: BMC Genomics. 2013 Dec 17;14:895. doi: 10.1186/1471-2164-14-895 (PMC3897883; doi:10.1186/1471-2164-14-895)
Supplement: Additional file 1: Table S1 — Genome coverage by sequence method for seven in-house sequenced SAG strains. [file 1471-2164-14-895-S1.docx]

Additional file 1, Table S1: Average coverage of 454 and Illumina sequencing for sequenced SAG strains.

| Strain | 454 sequence coverage | | | Illumina sequence coverage | | |
| --- | --- | --- | --- | --- | --- | --- |
|  | Total aligned bases | Genome size | Avg Coverage | Total aligned bases | Genome size | Avg Coverage |
| *S. anginosus* subsp. *whileyi* C238 | 76,225,246 | 2,233,640 | 34.1 | 618,678,784 | 2,233,640 | 277.0 |
| *S. anginosus* subsp. *anginosus* C1051 | 75,584,524 | 1,911,706 | 39.5 | 319,222,420 | 1,911,706 | 167.0 |
| *S. constellatus** C232 | 68,091,200 | 1,935,414 | 35.2 | NA | NA | NA |
| *S. constellatus* C818 | 100,324,354 | 1,935,662 | 51.8 | 310,392,892 | 1,935,662 | 160.4 |
| *S. constellatus* C1050 | 52,265,798 | 1,991,156 | 26.2 | 280,073,224 | 1,991,156 | 140.7 |
| *S. intermedius* B196 | 41,209,698 | 1,996,214 | 20.6 | 171,839,116 | 1,996,214 | 86.1 |
| *S. intermedius* C270 | 54,208,950 | 1,960,728 | 27.6 | 243,954,452 | 1,960,728 | 124.4 |

*All *S. constellatus* in this table are *S. constellatus* subsp. *pharyngis*; NA, Illumina sequencing not done for SCP C232.
