# Supplementary material for: Phylogenetic relationship and virulence inference of Streptococcus Anginosus Group: curated annotation and whole-genome comparative analysis support distinct species designation
Source: BMC Genomics. 2013 Dec 17;14:895. doi: 10.1186/1471-2164-14-895 (PMC3897883; doi:10.1186/1471-2164-14-895)
Supplement: Additional file 2: Figure S1 — MAUVE analysis of SAG genomes. A multiple alignment of SA, SI and SC to highlight accessory regions and potential synteny within the genomes. MAUVE 2.3.1 was used with default settings with and a reference genome for each species; SAW C238 (SA), SCC C232 (SC) and SI B196 (SI). [file 1471-2164-14-895-S2.pdf]

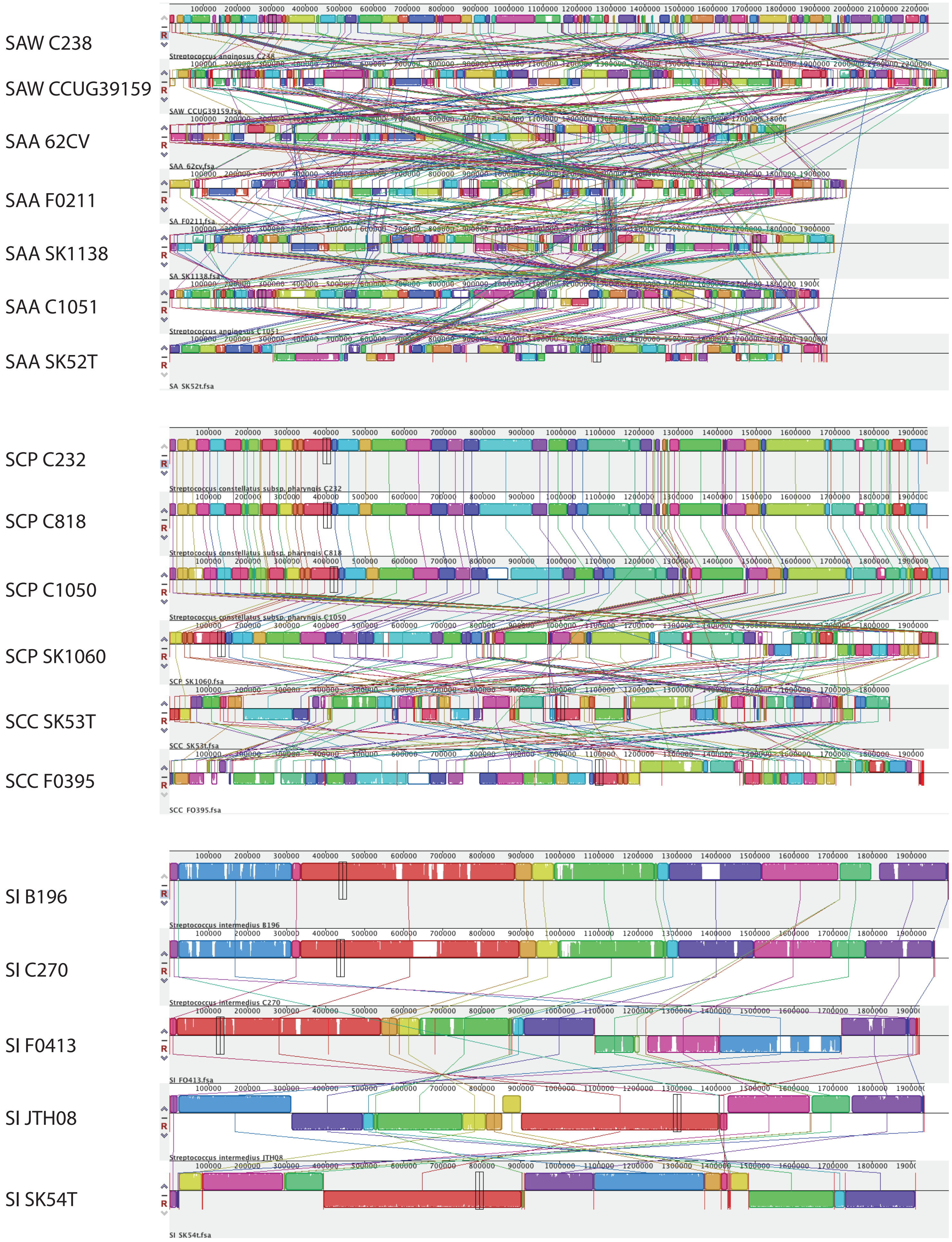
SAW C238
Streptococcus anginosus C238
SAW CCUG39159
SAW CCUG39159.fsa
SAA 62CV
SAA 62cv.fsa
SAA F0211
SA F0211.fsa
SAA SK1138
SA SK1138.fsa
SAA C1051
Streptococcus anginosus C1051
SAA SK52T
SA SK52t.fsa
SCP C232
Streptococcus constellatus subsp. pharyngis C232
SCP C818
Streptococcus constellatus subsp. pharyngis C818
SCP C1050
Streptococcus constellatus subsp. pharyngis C1050
SCP SK1060
SCP SK1060.fsa
SCC SK53T
SCC SK53t.fsa
SCC F0395
SCC FO395.fsa
SI B196
Streptococcus intermedius B196
SI C270
Streptococcus intermedius C270
SI F0413
SI FO413.fsa
SI JTH08
Streptococcus intermedius JTH08
SI SK54T
SI SK54t.fsa
