# Supplementary figures and images for: Phylogenetic relationship and virulence inference of Streptococcus Anginosus Group: curated annotation and whole-genome comparative analysis support distinct species designation
Source: BMC Genomics. 2013 Dec 17;14:895. doi: 10.1186/1471-2164-14-895 (PMC3897883; doi:10.1186/1471-2164-14-895)

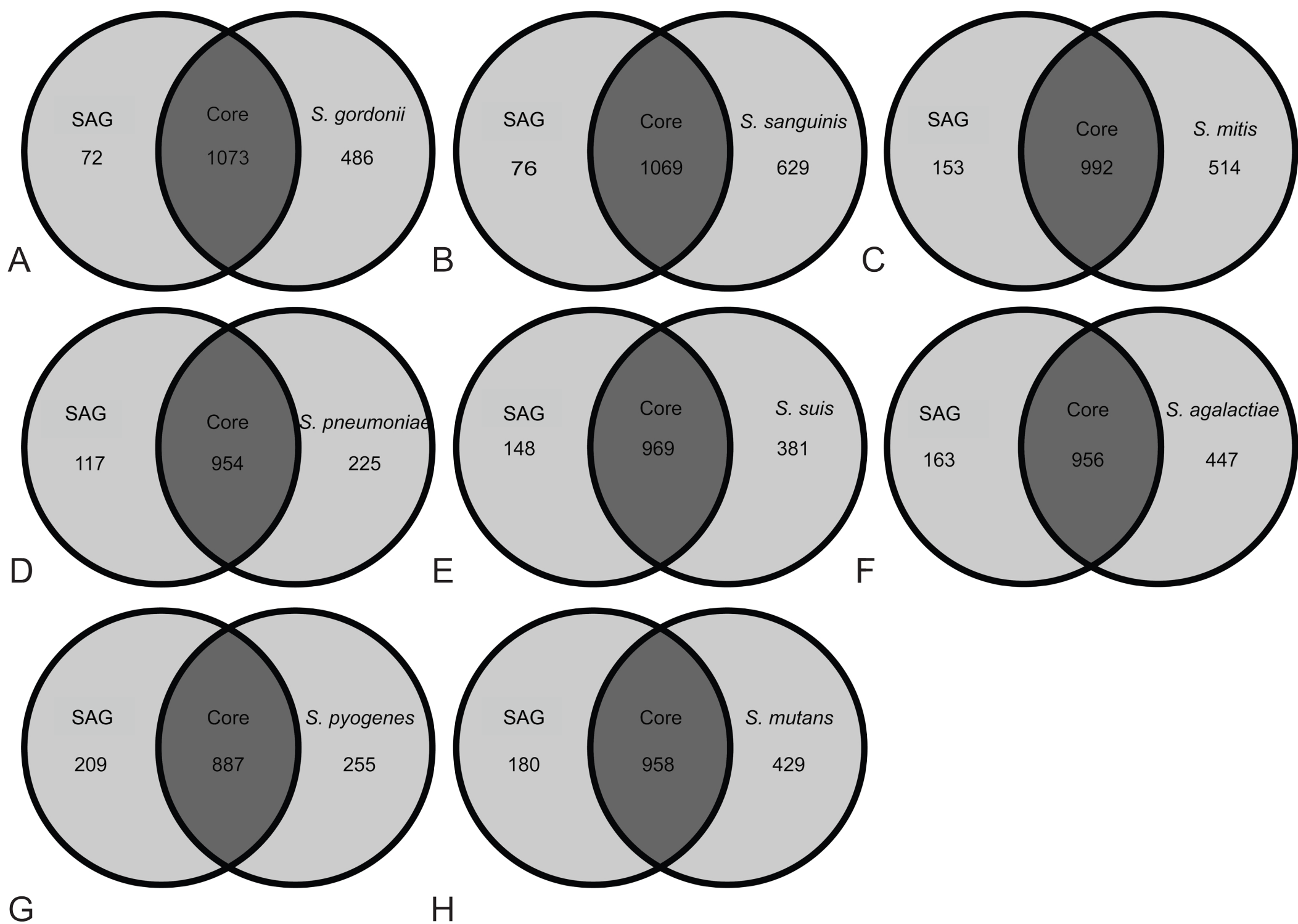

Supplement: Additional file 4: Figure S2 — Genomic content analysis comparison of SAG to other clinically important species within the genus Streptococcus. A) S. suis, B) S. pyogenes, C) S. agalactiae, D) S. pneumoniae, E) S. mutans F) S. mitis, G) S. sanguinis, H) S. gordonii. Numbers within circles correspond to number of CDSs that are conserved within the strains analyzed or that are unique to each strain analyzed. [file 1471-2164-14-895-S4.pdf]
