# Supplementary material for: Phylogenetic relationship and virulence inference of Streptococcus Anginosus Group: curated annotation and whole-genome comparative analysis support distinct species designation
Source: BMC Genomics. 2013 Dec 17;14:895. doi: 10.1186/1471-2164-14-895 (PMC3897883; doi:10.1186/1471-2164-14-895)
Supplement: Additional file 5: Figure S3 — Clusters of orthologous group (COG) analysis of 66 Streptococcus genomes. The phylogenetic tree was constructed using an in-house core-SNP pipeline as described previously. This Figure is a small portion of the overall comparison of all orthologs representing COGs present or absent in SAG while absent or present within the majority of other analysed Streptococcus. The COG map was created using OrthoMCL, COG functional categories are shown on the far left portion of the Figure. The Streptococcus species used for analysis are listed in Additional file 2, Table S2. [file 1471-2164-14-895-S5.pdf]

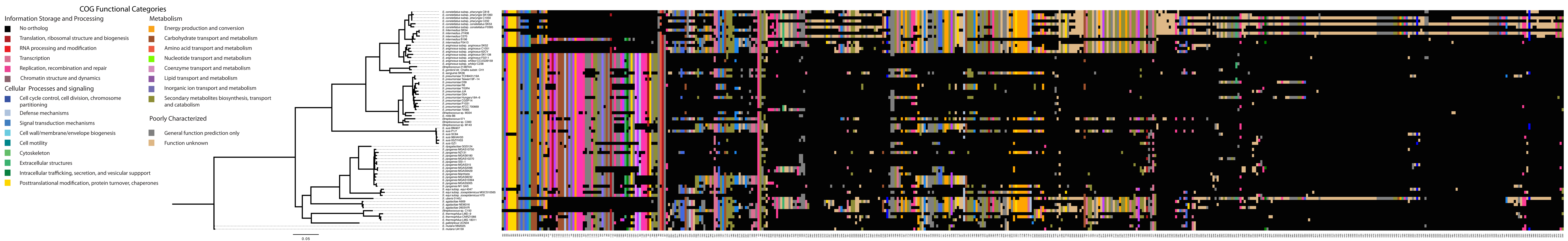
COG Functional Categories
Information Storage and Processing
No ortholog
Translation, ribosomal structure and biogenesis
RNA processing and modification
Transcription
Replication, recombination and repair
Chromatin structure and dynamics
Cellular Processes and signaling
Cell cycle control, cell division, chromosome partitioning
Defense mechanisms
Signal transduction mechanisms
Cell wall/membrane/envelope biogenesis
Cell motility
Cytoskeleton
Extracellular structures
Intracellular trafficking, secretion, and vesicular suppport
Posttranslational modification, protein turnover, chaperones
Metabolism
Energy production and conversion
Carbohydrate transport and metabolism
Amino acid transport and metabolism
Nucleotide transport and metabolism
Coenzyme transport and metabolism
Lipid transport and metabolism
Inorganic ion transport and metabolism
Secondary metabolites biosynthesis, transport and catabolism
Poorly Characterized
General function prediction only
Function unknown
0.05
