# Supplementary material for: Phylogenetic relationship and virulence inference of Streptococcus Anginosus Group: curated annotation and whole-genome comparative analysis support distinct species designation
Source: BMC Genomics. 2013 Dec 17;14:895. doi: 10.1186/1471-2164-14-895 (PMC3897883; doi:10.1186/1471-2164-14-895)
Supplement: Additional file 6: Table S3 — Comparison of gene content of SAG species to S. gordonii str. Challis substr. CH1 and S. sanguinis SK36. [file 1471-2164-14-895-S6.docx]

Additional file 6, Table S3: Comparison of gene content of SAG species to *S. gordonii* str. Challis substr. CH1 and *S. sanguinis* SK36.

| Species | Core genes | # of SAG core genes not found | Total SAG genes analyzed | % of SAG core genes found |
| --- | --- | --- | --- | --- |
| SI^a^ vs *S. gordonii* | 1283 | 153 | 1436 | 89.35 |
| SA^b^ vs *S. gordonii* | 1188 | 134 | 1322 | 89.86 |
| SCP^c^ vs *S. gordonii* | 1200 | 215 | 1415 | 84.80 |
| SCC^d^  vs *S. gordonii* | 1273 | 400 | 1673 | 76.10 |
| SI vs *S. sanguinis* | 1254 | 182 | 1436 | 87.39 |
| SA vs *S. sanguinis* | 1197 | 125 | 1322 | 90.54 |
| SCP vs *S. sanguinis* | 1205 | 210 | 1415 | 85.16 |
| SCC vs *S. sanguinis* | 1290 | 383 | 1673 | 77.11 |
| SCC vs SCP | 1366 | 132 | 1498 | 91.19 |

* This table was constructed using results from OrthoMCL; ^a)^ *S. intermedius*; ^b)^ *S. anginosus*; ^c)^ *S. constellatus* subsp. *pharyngis*; ^d)^ *S. constellatus* subsp. *constellatus.*
