# Supplementary material for: Phylogenetic relationship and virulence inference of Streptococcus Anginosus Group: curated annotation and whole-genome comparative analysis support distinct species designation
Source: BMC Genomics. 2013 Dec 17;14:895. doi: 10.1186/1471-2164-14-895 (PMC3897883; doi:10.1186/1471-2164-14-895)
Supplement: Additional file 7: Table S4 — Genes unique to sequenced SC, SI or SA. [file 1471-2164-14-895-S7.docx]

Additional File 7, Table S4: Genes unique to sequenced *S. constellatus*, *S. intermedius* or *S. anginosus.*

| Organism | Locus name | Length (AA) | Gene product | G + C content | COG # |
| --- | --- | --- | --- | --- | --- |
| *S. constellatus* | SCRE_0231 | 219 | hypothetical protein | 36.36 | 6334 |
| *S. constellatus* | SCRE_0261 | 277 | hypothetical protein | 32.61 | 6335 |
| *S. constellatus* | SCRE_0339 | 119 | hypothetical protein | 33.06 | 6336 |
| *S. constellatus* | SCRE_0340 | 583 | hypothetical protein | 33.22 | 6337 |
| *S. constellatus* | SCRE_0453 | 41 | hypothetical protein | 34.13 | 6338 |
| *S. constellatus* | SCRE_0460 | 178 | hypothetical protein | 27.37 | 6340 |
| *S. constellatus* | SCRE_0461 | 325 | hypothetical protein | 30.67 | 6341 |
| *S. constellatus* | SCRE_0462 | 256 | conserved hypothetical protein | 40.47 | 6342 |
| *S. constellatus* | SCRE_0854 | 240 | hypothetical protein | 28.49 | 5576 |
| *S. constellatus* | SCRE_0856 | 289 | hypothetical protein | 32.18 | 4804 |
| *S. constellatus* | SCRE_1112 | 1123 | hypothetical protein | 27.67 | 4466 |
| *S. constellatus* | SCRE_1149 | 107 | hypothetical protein | 32.41 | 5586 |
| *S. constellatus* | SCRE_1154 | 67 | hypothetical protein | 35.78 | 5589 |
| *S. constellatus* | SCRE_1158 | 71 | hypothetical protein | 34.72 | 5590 |
| *S. constellatus* | SCRE_1160 | 328 | conserved hypothetical protein | 38.50 | 5592 |
| *S. constellatus* | SCRE_1162 | 170 | hypothetical protein | 33.72 | 5593 |
| *S. constellatus* | SCRE_1163 | 197 | hypothetical protein | 34.68 | 5594 |
| *S. constellatus* | SCRE_1164 | 98 | hypothetical protein | 33.00 | 5595 |
| *S. constellatus* | SCRE_1165 | 93 | hypothetical protein | 32.98 | 5596 |
| *S. constellatus* | SCRE_1166 | 941 | hypothetical protein | 35.99 | 5597 |
| *S. constellatus* | SCRE_1167 | 224 | hypothetical protein | 32.44 | 5598 |
| *S. constellatus* | SCRE_1168 | 937 | putative phage protein | 37.92 | 5599 |
| *S. constellatus* | SCRE_1183 | 216 | hypothetical protein | 35.64 | 5608 |
| *S. constellatus* | SCRE_1189 | 166 | hypothetical protein | 31.94 | 5612 |
| *S. constellatus* | SCRE_1193 | 224 | conserved hypothetical protein | 36.15 | 5613 |
| *S. constellatus* | SCRE_1194 | 48 | hypothetical protein | 35.37 | 5614 |
| *S. constellatus* | SCRE_1199 | 203 | hypothetical protein | 35.13 | 5617 |
| *S. constellatus* | SCRE_1200 | 304 | hypothetical protein | 33.22 | 5142 |
| *S. constellatus* | SCRE_1201 | 224 | hypothetical protein | 31.85 | 5143 |
| *S. constellatus* | SCRE_1202 | 64 | hypothetical protein | 31.28 | 5618 |
| *S. constellatus* | SCRE_1203 | 753 | hypothetical protein | 34.26 | 5619 |
| *S. constellatus* | SCRE_1204 | 463 | hypothetical protein | 32.47 | 5620 |
| *S. constellatus* | SCRE_1206 | 378 | putative DNA methylase | 33.25 | 5622 |
| *S. constellatus* | SCRE_1209 | 61 | hypothetical protein | 29.03 | 5623 |
| *S. constellatus* | SCRE_1269 | 36 | hypothetical protein | 29.73 | 5631 |
| *S. constellatus* | SCRE_1270 | 145 | hypothetical protein | 27.17 | 5632 |
| *S. constellatus* | SCRE_1397 | 64 | hypothetical protein | 35.38 | 5634 |
| *S. constellatus* | SCRE_1401 | 195 | hypothetical protein | 30.61 | 5636 |
| *S. constellatus* | SCRE_1525 | 131 | conserved hypothetical protein | 30.56 | 5638 |
| *S. constellatus* | SCRE_1529 | 56 | hypothetical protein | 43.27 | 5639 |
| *S. constellatus* | SCRE_1710 | 62 | hypothetical protein | 34.39 | 5641 |
| *S. constellatus* | SCRE_1788 | 282 | hypothetical protein | 32.86 | 5643 |
| *S. anginosus* | SANR_0909 | 151 | hypothetical protein | 38.38 | 6257 |
| *S. anginosus* | SANR_1023 | 265 | oxidoreductase | 47.37 | 6258 |
| *S. anginosus* | SANR_1405 | 273 | hypothetical protein | 37.96 | 3843 |
| *S. anginosus* | SANR_1459 | 152 | hypothetical protein | 32.9 | 5331 |
| *S. intermedius* | SIR_0179 | 743 | hypothetical protein | 44.76 | 5015 |
| *S. intermedius* | SIR_0297 | 381 | hypothetical protein | 37.09 | 7078 |
| *S. intermedius* | SIR_0962 | 116 | hypothetical protein | 38.18 | 5358 |
| *S. intermedius* | SIR_0185 | 107 | hypothetical protein | 30.86 | 6020 |
| *S. intermedius* | SIR_0189 | 111 | hypothetical protein | 28.57 | 5504 |
| *S. intermedius* | SIR_0195 | 104 | hypothetical protein | 25.71 | 5423 |
| *S. intermedius* | SIR_0473 | 133 | hypothetical protein | 38.31 | 4680 |
| *S. intermedius* | SIR_0833 | 180 | hypothetical protein | 32.6 | 6279 |
| *S. intermedius* | SIR_0963 | 215 | hypothetical protein | 26.54 | 4967 |
| *S. intermedius* | SIR_0964 | 242 | hypothetical protein | 27.43 | 4968 |
| *S. intermedius* | SIR_0974 | 205 | putative peptidase E | 31.23 | 4154 |
| *S. intermedius* | SIR_1073 | 101 | hypothetical protein | 42.16 | 3795 |
| *S. intermedius* | SIR_1188 | 321 | conserved hypothetical protein | 33.54 | 4158 |
| *S. intermedius* | SIR_1490 | 116 | hypothetical protein | 36.47 | 4973 |
