# Supplementary material for: Phylogenetic relationship and virulence inference of Streptococcus Anginosus Group: curated annotation and whole-genome comparative analysis support distinct species designation
Source: BMC Genomics. 2013 Dec 17;14:895. doi: 10.1186/1471-2164-14-895 (PMC3897883; doi:10.1186/1471-2164-14-895)
Supplement: Additional file 9: Table S6 — Streptococcus constellatus genes with a match to the virulence gene database. [file 1471-2164-14-895-S9.docx]

Additional File 9, Table S6: *Streptococcus constellatus* genes with a match to the virulence gene database.

| Database reference | Region | | | PID^a^ | % HSP^b^ | Gene Name | Description |
| --- | --- | --- | --- | --- | --- | --- | --- |
| **gi\|32812823*** | **SCRE_0199** | **SCR2_0199** | **SCI_0219** | **97** | **100.0** | ***gap*dh** | **glyceraldehyde 3-phosphate dehydrogenase** |
| **gi\|16151617** | **SCRE_0912** | **SCR2_0912** | **SCI_0984** | **96** | **100.0** | ***eno*** | **alpha-enolas** |
| **gi\|1881547** | **SCRE_1141** | **SCR2_1141** | **SCI_1200** | **92** | **98.9** | ***cps19f*O** | **capsular polysaccharide biosynthesis operon** |
| **gi\|1881546** | **SCRE_1127** | **SCR2_1127** | **SCI_1186** | **91** | **99.4** | ***cps19f*N** | **capsular polysaccharide biosynthesis operon** |
| **gi\|1881544** | **SCRE_1129** | **SCR2_1129** | **SCI_1188** | **90** | **99.7** | ***cps19f*L** | **capsular polysaccharide biosynthesis operon** |
| **gi\|1881545** | **SCRE_1128** | **SCR2_1128** | **SCI_1187** | **90** | **99.5** | ***cps19f*M** | **capsular polysaccharide biosynthesis operon** |
| **VFG0964** | **SCRE_1707** | **SCR2_1707** | **SCI_1751** | **86** | **97.4** | ***has*C** | **UDP-glucose pyrophosphorylase** |
| **VFG1359** | **SCRE_1630** | **SCR2_1630** | **SCI_1674** | **83** | **99.7** | ***psa*A** | **manganese ABC transporter** |
| VFG0977 | SCRE_0367 | SCR2_0367 | SCI_0387 | 83 | 68.5 | *sagA* | Streptolysin-S operon |
| **gi\|253559383** | **SCRE_0559** | **SCR2_0559** | **SCI_0579** | **79** | **99.7** | ***sil*E** | **Streptococcal invasion locus** |
| **VFG0959** | **SCRE_0627** | **SCR2_0627** | **SCI_0647** | **73** | **99.8** | ***fbp*** | **Fibronectin binding protein** |
| VFG1364 | SCRE_1598 | SCR2_1598 | SCI_1642 | 72 | 99.8 | *hyl* | hyaluronidase |
| gi\|8825620 | SCRE_0369 | SCR2_0369 | SCI_0389 | 68 | 99.2 | *sagC* | Streptolysin-S operon |
| gi\|3283389 | SCRE_0368 | SCR2_0368 | SCI_0388 | 64 | 99.4 | *sagB* | Streptolysin-S operon |
| **VFG1330** | **SCRE_0739** | **SCR2_0739** | **SCI_0759** | **62** | **99.7** | ***lmb*** | **laminin-binding surface protein** |
| **gi\|22797659** | **SCRE_1623** | **SCR2_1623** | **SCI_1667** | **57** | **86.6** | ***pul*A** | **pullulanase** |
| **gi\|253559382** | **SCRE_0560** | **SCR2_0560** | **SCI_0580** | **55** | **96.5** | ***sil*D** | **Streptococcal invasion locus** |
| **gi\|253559378** | **SCRE_0563** | **SCR2_0563** | **SCI_0583** | **49** | **99.6** | ***sil*A** | **Streptococcal invasion locus** |
| **gi\|6002654** | **SCRE_1473** | **SCR2_1473** | **SCI_1516** | **46** | **99.1** | ***csr*R** | **response regulato** |
| **gi\|4886774** | **SCRE_0303** | **SCR2_0303** | **SCI_0323** | **42** | **86.4** | ***cyl*Z** | **cyl gene cluster** |
| **gi\|11245963** | **SCRE_0526** | **SCR2_0526** | **SCI_0546** | **42** | **87.1** | ***sal*X** | **salivaricin A gene** |
| **gi\|253559379** | **SCRE_0562** | **SCR2_0562** | **SCI_0582** | **41** | **84.0** | ***sil*B** | **Streptococcal invasion locus** |
| **gi\|253559380** | **SCRE_0561** | **SCR2_0561** | **SCI_0581** | **41** | **55.1** | ***sil*CR** | **Streptococcal invasion locus** |
| gi\|1161270 | SCRE_0264 | SCR2_0264 | SCI_0284 | 39 | 96.7 | *spx*B | pyruvate oxidase |
| **gi\|4886772** | **SCRE_0300** | **SCR2_0300** | **SCI_0320** | **37** | **95.5** | ***cyl*G** | **cyl gene cluster** |
| gi\|209559120 | SCRE_0370 | SCR2_0370 | SCI_0390 | 80 | 100.0 | sagD | Streptolysin S biosynthesis protein |
| gi\|209559121 | SCRE_0371 | SCR2_0371 | SCI_0391 | 46 | 98.7 | *sagE* | Streptolysin S self-immunity protein |
| gi\|209559122 | SCRE_0372 | SCR2_0372 | SCI_0392 | 39 | 91.4 | *sagF* | Streptolysin S biosynthesis protein |
| gi\|209559123 | SCRE_0373 | SCR2_0373 | SCI_0393 | 70 | 100.0 | *sagG* | Streptolysin S export ATP-binding protein |
| gi\|209559124 | SCRE_0374 | SCR2_0374 | SCI_0394 | 73 | 99.7 | *sagH* | Streptolysin S export transmembrane protein |
| gi\|209559125 | SCRE_0375 | SCR2_0375 | SCI_0395 | 69 | 99.2 | *sagI* | Streptolysin S export transmembrane protein |
| gi\|209559600 | SCRE_0964 | SCR2_0964 | SCI_1023 | 47 | 89.2 | *inl*A | internalin |
| VFG0963 | NA | NA | SCI_0838 | 43 | 99.5 | *has*B | UDP-glucose 6-dehydrogenase |

***Bolded items are conserved in all sequenced SAG strains from this study**

^a^Percent coverage of protein from SAG compared to best match in NCBI database using BlastX

^b^PID is equal to the percent protein identity for the best match using BlastX from NCBI
