# Supplementary material for: Phylogenetic relationship and virulence inference of Streptococcus Anginosus Group: curated annotation and whole-genome comparative analysis support distinct species designation
Source: BMC Genomics. 2013 Dec 17;14:895. doi: 10.1186/1471-2164-14-895 (PMC3897883; doi:10.1186/1471-2164-14-895)
Supplement: Additional file 10: Table S7 — S. intermedius genes with a match to the virulence gene database. [file 1471-2164-14-895-S10.docx]

Additional File 10, Table S7: *Streptococcus intermedius* genes with a match to the virulence gene database.

| Database reference | Region | | PID^a^ | % HSP^b^ | Gene Name | Description |
| --- | --- | --- | --- | --- | --- | --- |
| **gi\|32812823** | **SIR_0213** | **SII_0201** | **98** | **100.0** | ***gap*dh** | **glyceraldehyde 3-phosphate dehydrogenase** |
| **gi\|16151617** | **SIR_0832** | **SII_0848** | **96** | **100.0** | ***eno*** | **alpha-enolas** |
| **gi\|1881546** | **SIR_0938** | **SII_0955** | **93** | **99.4** | ***cps19f*N** | **capsular polysaccharide biosynthesis operon** |
| **gi\|1881547** | **SIR_0958** | **SII_0975** | **91** | **98.9** | ***cps19f*O** | **capsular polysaccharide biosynthesis operon** |
| **gi\|1881544** | **SIR_0940** | **SII_0957** | **90** | **99.7** | ***cps19f*L** | **capsular polysaccharide biosynthesis operon** |
| **gi\|1881545** | **SIR_0939** | **SII_0956** | **90** | **99.5** | ***cps19f*M** | **capsular polysaccharide biosynthesis operon** |
| **VFG0964** | **SIR_1692** | **SII_1683** | **87** | **98.0** | ***has*C** | **UDP-glucose pyrophosphorylase** |
| **VFG1359** | **SIR_1611** | **SII_1597** | **83** | **100.0** | ***psa*A** | **manganese ABC transporter** |
| **gi\|253559383** | **SIR_1155** | **SII_1176** | **80** | **99.7** | ***sil*E** | **Streptococcal invasion locus** |
| **VFG0959** | **SIR_0464** | **SII_0448** | **73** | **99.8** | ***fbp*** | **Fibronectin binding protein** |
| VFG1364 | SIR_1547 | SII_1533 | 73 | 99.8 | *hyl* | hyaluronidase |
| VFG1366 | SIR_1786 | SII_1753 | 71 | 99.2 | *cps4*B | capsular polysaccharide biosynthesis protein |
| VFG1378 | SIR_0015 | SII_0015 | 71 | 85.5 | *nan*A | Sialidase A precursor |
| VFG1369 | SIR_1781 | SII_1749 | 38 | 81.1 | *cps4*E | capsular polysaccharide biosynthesis protein |
| VFG1368 | SIR_1784 | SII_1751 | 66 | 97.8 | *cps4*D | capsular polysaccharide biosynthesis protein |
| VFG1365 | SIR_1787 | SII_1754 | 63 | 99.0 | *cps4*A | capsular polysaccharide biosynthesis protein |
| VFG1367 | SIR_1785 | SII_1752 | 62 | 99.6 | *cps4*C | capsular polysaccharide biosynthesis protein |
| **VFG1330** | **SIR_0653** | **SII_0681** | **61** | **99.7** | ***lmb*** | **laminin-binding surface protein** |
| VFG1374 | SIR_1774 | SII_1750 | 58 | 98.3 | *cps19f*j | capsular polysaccharide biosynthesis operon |
| **gi\|22797659** | **SIR_1606** | **SII_1592** | **57** | **86.6** | ***pul*A** | **pullulanase** |
| **gi\|253559382** | **SIR_1154** | **SII_1175** | **56** | **99.8** | ***sil*D** | **Streptococcal invasion locus** |
| gi\|257348146 | SIR_1773 | SII_1742 | 55 | 73.8 | *pav*B | adhesion |
| VFG1363 | SIR_0115 | SII_0120 | 53 | 88.3 | *ply* | Pneumolysin |
| **gi\|253559378** | **SIR_1151** | **SII_1172** | **50** | **99.2** | ***sil*A** | **Streptococcal invasion locus** |
| **gi\|6002654** | **SIR_0348** | **SII_0335** | **45** | **99.1** | ***csr*R** | **response regulator** |
| gi\|253559384 | SIR_1142 | SII_1163 | 43 | 52.0 | *blp*M | Streptococcal invasion locus |
| **gi\|4886774** | **SIR_1434** | **SII_1421** | **42** | **86.4** | ***cyl*Z** | **cyl gene cluster** |
| **gi\|11245963** | **SIR_1187** | **SII_1212** | **41** | **87.1** | ***sal*X** | **salivaricin A** |
| **gi\|253559379** | **SIR_1152** | **SII_1173** | **39** | **84.0** | ***Sil*B** | **Streptococcal invasion locus** |
| gi\|154432982 | SIR_0494 | SII_0084 | 41 | 86.5 | *srt*C | RlrA pilus operon |
| **gi\|4886775** | **SIR_1192** | **SII_1217** | **37** | **87.1** | ***cyl*G** | **cyl gene cluster** |
| gi\|4886772 | SIR_1437 | SII_1424 | 38 | 95.5 | *cyl*A | cyl gene cluster |
| **gi\|253559380** | **SIR_1153** | **SII_1174** | **37** | **70.0** | ***sil*CR** | **Streptococcal invasion locus** |
| gi\|75993664 | SIR_1402 | NA | 38 | 67.5 | *scp*C | chemokine protease C |

***Bolded items are conserved in all sequenced SAG strains from this study**

^a^Percent coverage of protein from SAG compared to best match in NCBI database using BlastX

^b^PID is equal to the percent protein identity for the best match using BlastX from NCBI
