# Supplementary material for: Phylogenetic relationship and virulence inference of Streptococcus Anginosus Group: curated annotation and whole-genome comparative analysis support distinct species designation
Source: BMC Genomics. 2013 Dec 17;14:895. doi: 10.1186/1471-2164-14-895 (PMC3897883; doi:10.1186/1471-2164-14-895)
Supplement: Additional file 11: Table S8 — S. anginosus genes with a match to the virulence gene database. [file 1471-2164-14-895-S11.docx]

Additional File 11, Table S8: *Streptococcus anginosus* genes with a match to the virulence gene database.

| db_ref | Region | | PID^a^ | % HSP^b^ | Gene Name | Description |
| --- | --- | --- | --- | --- | --- | --- |
| **gi\|32812823*** | **SAIN_1627** | **SANR_1894** | **97** | **100.0** | ***gap*dh** | **glyceraldehyde 3-phosphate dehydrogenase** |
| **gi\|16151617** | **SAIN_0997** | **SANR_1074** | **95** | **100.0** | ***eno*** | **alpha-enolas** |
| **gi\|1881547** | **SAIN_0666** | **SANR_0675** | **93** | **98.9** | ***cps19f*O** | **capsular polysaccharide biosynthesis** |
| **gi\|1881546** | **SAIN_0657** | **SANR_0666** | **91** | **99.4** | ***cps19f*N** | **capsular polysaccharide biosynthesis** |
| **gi\|1881544** | **SAIN_0655** | **SANR_0664** | **90** | **99.7** | ***cps19f*L** | **capsular polysaccharide biosynthesis** |
| **gi\|1881545** | **SAIN_0656** | **SANR_0665** | **90** | **99.5** | ***cps19f*M** | **capsular polysaccharide biosynthesis** |
| **VFG0964** | **SAIN_1619** | **SANR_1886** | **86** | **97.4** | ***has*C** | **UDP-glucose pyrophosphorylase** |
| **VFG1359** | **SAIN_0244** | **SANR_0284** | **83** | **100.0** | ***psa*A** | **manganese ABC transporter** |
| **gi\|253559383** | **SAIN_0537** | **SANR_0554** | **80** | **99.7** | ***sil*E** | **Streptococcal invasion locus** |
| **VFG0959** | **SAIN_0616** | **SANR_0625** | **75** | **99.8** | ***fbp*** | **Fibronectin binding protein** |
| **VFG1330** | **SAIN_1064** | **SANR_1155** | **62** | **99.7** | ***lmb*** | **laminin-binding surface protein** |
| **gi\|22797659** | **SAIN_1534** | **SANR_1773** | **59** | **81.3** | ***pul*A** | **pullulanase** |
| **gi\|253559382** | **SAIN_0538** | **SANR_0555** | **54** | **99.3** | ***sil*D** | **Streptococcal invasion locus** |
| **gi\|253559378** | **SAIN_0541** | **SANR_1817** | **52** | **98.4** | ***sil*A** | **Streptococcal invasion locus** |
| **gi\|6002654** | **SAIN_1370** | **SANR_1594** | **46** | **99.1** | ***csr*R** | **response regulator** |
| **gi\|11245963** | **SAIN_1156** | **SANR_0814** | **44** | **88.6** | ***sal*X** | **salivaricin A gene** |
| gi\|253559384 | SAIN_0549 | SANR_0567 | 43 | 51.3 | *blp*M | Streptococcal invasion locus |
| **gi\|4886774** | **SAIN_0266** | **SANR_0306** | **42** | **86.4** | ***cyl*Z** | **cyl gene cluster** |
| **gi\|253559379** | **SAIN_0540** | **SANR_0557** | **41** | **84.0** | ***sil*B** | **Streptococcal invasion locus** |
| **gi\|253559380** | **SAIN_0539** | **SANR_0556** | **41** | **55.1** | ***sil*CR** | **Streptococcal invasion locus** |
| **gi\|4886772** | **SAIN_0263** | **SANR_0303** | **38** | **95.1** | ***cyl*G** | **cyl gene cluster** |
| gi\|8825620 | NA | SANR_0369 | 68 | 99.2 | *sagC* | Streptolysin-S operon |
| gi\|3283389 | NA | SANR_0368 | 64 | 99.4 | *sagB* | Streptolysin-S operon |
| VFG1364 | NA | SANR_1718 | 63 | 97.0 | *hyl* | hyaluronidase |
| VFG0977 | NA | SANR_0367 | 83 | 68.5 | *sagA* | Streptolysin-S operon |
| gi\|1161270 | NA | SANR_1810 | 39 | 96.7 | *spx*B | pyruvate oxidase |
| gi\|209559120 | NA | SANR_0370 | 80 | 100.0 | *sagD* | Streptolysin S biosynthesis protein |
| gi\|209559121 | NA | SANR_0371 | 46 | 98.2 | *sagE* | Streptolysin S self-immunity protein |
| gi\|209559122 | NA | SANR_0372 | 37 | 91.4 | *sagF* | Streptolysin S biosynthesis protein |
| gi\|209559123 | NA | SANR_0373 | 72 | 100.0 | *sagG* | Streptolysin S export ATP-binding protein |
| gi\|209559124 | NA | SANR_0374 | 73 | 99.7 | *sagH* | Streptolysin S export transmembrane protein |
| gi\|209559125 | NA | SANR_0375 | 69 | 99.2 | *sagI* | Streptolysin S export transmembrane protein |
| gi\|209559600 | NA | SANR_0893 | 47 | 89.2 | *inlA* | Internalin A protein |
| gi\|1161270 | SAIN_1574 | NA | 97 | 100.0 | spxB | pyruvate oxidase |
| VFG1366 | SAIN_1729 | NA | 71 | 100.0 | *cps4*B | capsular polysaccharide biosynthesis protein |
| VFG1368 | SAIN_1727 | NA | 68 | 97.4 | *cps4*D | capsular polysaccharide biosynthesis protein |
| gi\|485275 | SAIN_1730 | NA | 64 | 98.6 | *cps19f*A | capsular polysaccharide biosynthesis operon |
| VFG1367 | SAIN_1728 | NA | 64 | 99.1 | *cps4*C | capsular polysaccharide biosynthesis protein |
| gi\|257348146 | SAIN_1710 | NA | 62 | 56.3 | *pav*B | adhesion |
| VFG1373 | SAIN_1479 | NA | 58 | 94.0 | *cpns4*I | capsular polysaccharide biosynthesis protein |
| gi\|485279 | SAIN_1726 | NA | 56 | 98.2 | *cps19f*E | capsular polysaccharide biosynthesis operon |
| gi\|4886775 | SAIN_0499 | NA | 38 | 87.4 | *cyl*A | cyl gene cluster |

***Bolded items are conserved in all sequenced SAG strains from this study**

^a^Percent coverage of protein from SAG compared to best match in NCBI database using BlastX

^b^PID is equal to the percent protein identity for the best match using BlastX from NCBI
