# Supplementary material for: Phylogenetic relationship and virulence inference of Streptococcus Anginosus Group: curated annotation and whole-genome comparative analysis support distinct species designation
Source: BMC Genomics. 2013 Dec 17;14:895. doi: 10.1186/1471-2164-14-895 (PMC3897883; doi:10.1186/1471-2164-14-895)
Supplement: Additional file 12: Table S9 — SAG LPxTG proteins. [file 1471-2164-14-895-S12.docx]

Additional File 12, Table S9: SAG LPxTG proteins.

| Strain present in | LPxTG | Loci^a^ | Protein Characteristics^b^ | PID^c^ | % HSP^d^ | Top Blast hit to NCBI protein database |
| --- | --- | --- | --- | --- | --- | --- |
| All SAG | LPSTG | SCRE_0485 | Nuclease/phosphatase | 95 | 100.0 | *S. anginosus* SK52 |
| All SAG | LPKTG | SCRE_0571 | Pullulanase | 99 | 100.0 | *S. anginosus* 62cv |
| All SAG | LPATG | SCRE_0820 | Chromosome segregation | 93 | 100.0 | *S. anginosus* SK52 |
| All SAG | LPSTG | SCRE_1355 | dipeptidase | 96 | 100.0 | *S. anginosus* 62cv |
| All SAG | LPSTG | SCRE_1623 | Pullulanase | 83 | 97.0 | *S. anginosus* F0211 |
| SI/SC/ SAW^f^ | LPQTG | SCRE_1598 | Hyaluronate lyase precursor | 100 | 100.0 | *S. constellatus* |
| SC^g^/SA^h^ | LPKTG | SCRE_0115 | Pilin isopeptide linkage domain | 85 | 82.5 | *S. anginosus* F0211 |
| SC/SA | LPQAG | SCRE_0528 | AA permease | 95 | 100.0 | *S. anginosus* 62cv |
| SC/ C238^i^ | LPATG | SCRE_0436 | Albumin binding | 62 | 99.1 | *S. canis* |
| C232^j^/C818^k^/SA | LPQTG | SCRE_0946 | Unknown | 93 | 100.0 | *S. anginosus* SK52 |
| C238, SI^l^ | LPRTG | SANR_1717 | Collagen binding | 36 | 105.0 | *S. sanguinis* SK1087 |
| SC | LPSTG | SCRE_0168 | Unknown | 52 | 80.5 | Streptococcus. sp 2_1_36FAA |
| ***SC*** | ***LPKTG*** | **SCRE_1214** | ***Collagen binding protein*** | ***NH*** | ***NH*** | ***NH*** |
| SC | LPNTG | SCRE_1625 | Collagen binding protein A | 62 | 28.0 | *S. pneumoniae* JJA |
| ***SC*** | ***LPKTG*** | **SCRE_1780** | ***Collagen binding protein A*** | ***38*** | ***134.4*** | ***Anaerococcus prevotii*** |
| SA | LPETG | SANR_1066 | 5'-nucleotidase | 99 | 100.0 | *S. anginosus* 62cv |
| SA | LPSTG | SAIN_1264 | Glycosyl hydrolase | 98 | 100.0 | *S. anginosus* 62cv |
| SA | LPQTG | SAIN_1475 | Cell wall surface | 98 | 100.0 | *S. anginosus* 62cv |
| SI | LPSTG | SIR_0015 | Neuraminidase | 99 | 100.0 | *S. intermedius* |
| SI | LPKTG | SIR_0113 | Unknown | 82 | 87.4 | *S. anginosus* 62CV |
| SI | LPSTG | SIR_1072 | Chintinase | 59 | 72.0 | *S. cristatus* 51100 |
| ***SI*** | ***LPQTG*** | **SIR_1549** | ***Collagen binding*** | ***78*** | ***86.3*** | ***Parvimonas micra 33270*** |
| SI | LPQTG | SIR_1675 | Cell surfacce antigen I/II | 91 | 100.0 | *S. intermedius* |
| SI | LPNTG | SIR_1773 | Fibronecting binding | 92 | 100.0 | *S. anginosus* 62cv |
| C1050^m^ | LPETG | SCI_0808 | Cell wall | 50 | 102.6 | *S. infantarius* BAA-102 |
| C1050 | LPTTG | SCI_0810 | Cell surface antigen I/II | 57 | 106.3 | *S. agalactiae* |
| C1051 | LPDTG | SAIN_0149 | Unknown | 99 | 100.0 | *S. anginosus* 62cv |
| C1051 | LPSTG | SAIN_0150 | Cell surface antigen | 89 | 182.6 | *S. anginosus* 62cv |
| C1051 | LPHTG | SAIN_0367 | Cell surface antigen | 57 | 101.5 | *S. oralis* 35037 |
| ***C1051*** | ***LPHTG*** | **SAIN_0368** | ***Unknown*** | ***36*** | ***272.6*** | ***S. vestibularis*** |
| C1051 | LPSTG | SAIN_0748 | Unknown | 92 | 155.2 | *S. anginosus* 62cv |
| ***C1051*** | ***LPHTG*** | **SAIN_0813** | ***Cell surface antigen*** | ***NH^p^*** | ***NH*** | ***NH*** |
| C1051 | LPHTG | SAIN_0814 | Cell surface antigen | 57 | 101.5 | *S. oralis* 35037 |
| ***C1051*** | ***LPHTG*** | ***SAIN_0815*** | ***Unknown*** | ***36*** | ***272.6*** | ***S. vestibularis F0396*** |
| C1051 | LPNTG | SAIN_1506 | Unknown | 83 | 254.4 | *S. infantis* SK1302 |
| C238 | LPNTG | SANR_2057 | Unknown | 98 | 99.6 | *S. anginosus* SK52 |
| ***C238*** | ***LPKTG*** | ***SANR_1190*** | ***Cell surface antigen*** | ***28*** | ***65.4*** | ***S. vestibularis F0396*** |
| C238 | LPNTG | SANR_1162 | Cell surface antigen | 85 | 99.3 | *S. dysgalactiae* 27957 |
| ***C238*** | ***LPKTG*** | ***SANR_1229*** | ***Unknown*** | ***39*** | ***110.0*** | ***S. sp oral F0418*** |
| C238 | LPQTG | SANR_1413 | Unknown | 56 | 102.3 | *S. anginosus* SK52 |
| C238 | LPQTG | SANR_1414 | Unknown | 74 | 102.6 | *S. anginosus* F0211 |
| C238 | LPSTG | SANR_1415 | Unknown | 88 | 100.0 | *S. anginosus* SK52 |
| ***C238*** | ***LPKTG*** | ***SANR_1842*** | ***Collagen binding*** | ***NH*** | ***NH*** | ***NH*** |
| C238 | LPNTG | SANR_1854 | Unknown | 99 | 100.0 | *S. pneumoniae* GA17545 |
| C270^n^ | LPMTG | SII_0082 | Collagen binding | NH | 216.9 | *S. intermedius* |
| C270 | LPKTG | SII_0083 | Fimbrial structural subunit | 82 | 94.7 | *S. intermedius* |
| B196^o^ | LPNTG | SIR_0080 | Unknown | 69 | 111.4 | *S. anginosus* F0211 |
| B196 | LPNTG | SIR_0081 | Collagen binding | 77 | 100.4 | *S. anginosus* F0211 |
| B196 | LPSTG | SIR_0493 | Collagen binding | 62 | 97.3 | *S. pyogenes* MGAS10394 |
| B196 | LPSTG | SIR_1805 | Collagen binding | 71 | 60.3 | *S. anginosus* 62cv |

^a^SAG gene loci, if in more than one strain listed as a representative for all, ^b^Protein characteristic taken from top blast hit, ^c^PID = percent protein identity,^d^%HSP; % of SAG protein length as compared to the high scoring sequence pair, ^e^C1051 = *S. anginosus* C1051, ^f^*S. anginosus* subsp. *whileyi*, ^g^*S. constellatus*, ^h^*S. anginosus*, ^i^*S. anginosus*, ^j^*S. constellatus* subsp. *pharyngis* C232, ^k^*S. constellatus* subsp. *pharyngis* C818, ^l^*S. intermedius*, ^m^*S*. *Constellatus* subsp. *pharyngis* C1050, ^n^*S. Intermedius* C270 ^o^*S. Intermedius* B196, ^p^NH= no hits to the NCBI protein database. ***All proteins that are in bold and italics have no match to any proteins in the NCBI database.***
