# Supplementary material for: Phylogenetic relationship and virulence inference of Streptococcus Anginosus Group: curated annotation and whole-genome comparative analysis support distinct species designation
Source: BMC Genomics. 2013 Dec 17;14:895. doi: 10.1186/1471-2164-14-895 (PMC3897883; doi:10.1186/1471-2164-14-895)
Supplement: Additional file 13: Table S10 — Summary of CRISPRs found in SAG. [file 1471-2164-14-895-S13.docx]

Additional file 13, Table S10: Summary of CRISPRs found in SAG.

| Organism | Strain | CRISPR Name | CRISPR Position^2^ | DR Length (nt) | # of spacers | Spacer Length (nt) | Accession # | CRISPR family | Closest BlastP result^1^ |
| --- | --- | --- | --- | --- | --- | --- | --- | --- | --- |
| SI | B196 | SintB196-1 | 768951- 770239 | 36 | 19 | 30 (29-30) | ZP_08013533 | Type II-A | SA 62CV |
| SA | 62CV | Sang62-1 | 1345376- 1345674 | 36 | 4 | 30 (29-30) | ZP_14325277 | Type II-A | *S. parasanguinis* F0449 |
| SA | C1051 | Sang1051-1 | 334380- 335924 | 37 | 21 | 34 (32-39) | ZP_08013533 | Unclassified (truncated) | *S. sanguinis* SK1060 |
|  |  | Sang1051-2 | 942073- 944023 | 36 | 29 | 30 (30-31) | ZP_16901008 | Type II-A | SA 62CV |
|  |  | Sang1051-3 | 1295388- 1295623 | 38 | 3 | 28 | NA* | NA | NA |
| SCC | SK53T | SconSK53-1  SconSK53-2 | 485169- 486260  767730-769016 | 36  32 | 16  19 | 30  34 (33-36) | ZP_15935419  ZP_16928827 | Type II-A  Type 1-C | S. oralis SK304  *S. sanguinis* SK355 |
| SA | SK52T | SangSK52-1 | 1471976- 1473530 | 36 | 23 | 30 (30-31) | NA | Type II-A (truncated) | NA |
| SA | SK1138 | SangSK1138-1 | 1055192- 1055755 | 36 | 8 | 30 | ZP_15935419 | Type II-A | S. oralis SK304 |
| SA | F0211 | SangF0211-1  SangF0211-2  SangF0211-3 | 1205723- 1207248  1781187- 1782839  1837094- 1837834 | 28  28  28 | 23  25  11 | 38 (32-38)  38 (31-38)  38(33-38) | NA  NA  ZP_15935419 | NA  NA  Type II-A | NA  NA  S. oralis SK304 |

^1^Best match in GenBank based on aa identity for Cas1 proteins using BlastP. *NA = CRISPR region that do not have any Cas1 proteins present. All WGS contigs were assembled and aligned to a reference sequence (SCP C232 for *S. constellatus*, SI B196 for *S. intermedius* and SA C1051 for *S. anginosus*) to provide a CRISPR position.
