# Supplementary material for: Phylogenetic relationship and virulence inference of Streptococcus Anginosus Group: curated annotation and whole-genome comparative analysis support distinct species designation
Source: BMC Genomics. 2013 Dec 17;14:895. doi: 10.1186/1471-2164-14-895 (PMC3897883; doi:10.1186/1471-2164-14-895)
Supplement: Additional file 15: Table S12 — Comparative analysis of essential competence genes from S. pneumoniae TIGR4. [file 1471-2164-14-895-S15.docx]

Additional file 15, Table S12: Comparative analysis of essential competence genes from *S. pneumoniae* TIGR4.

| *Gene name* | *S. pneumoniae* TIGR4 locus^1^ | GenBank Accession # | Length of protein | C232 locus | HSP^2^ length | PID^3^ |
| --- | --- | --- | --- | --- | --- | --- |
| *com*X1  *com*X2 | SP_0014  SP_2006 | AAK74207.1  NP_346433.1 | 159  159 | SCRE_0016  SCRE_0287  SCRE_1735 | 160  160  160 | 53.0  53.0  53.0 |
| *com*W | SP_0018 | NP_344570.1 | 80 | _ | _ | _ |
| *com*A | SP_0042 | NP_344591.1 | 717 | SCRE_0559 | 713 | 65.1 |
| *com*B | SP_0043 | NP_344592.1 | 449 | SCRE_0560 | 442 | 33.9 |
| *com*E | SP_2235 | NP_346642.1 | 250 | SCRE_1893 | 245 | 70.6 |
| *com*D | SP_2236 | NP_346643.1 | 441 | SCRE_1894 | 376 | 48.1 |
| *com*C | SP_2237 | NP_346644.1 | 41 | SCRE_1895 | 48 | 35.4 |
| *cel*A/*com*EA* | SP_0954 | NP_345435.1 | 216 | SCRE_0788 | NA | NA |
| *cel*B/*com*EC | SP_0955 | NP_345436.1 | 746 | SCRE_0789 | 747 | 53.9 |
| *coi*A | SP_0978 | NP_345459.1 | 317 | SCRE_0592 | 314 | 53.2 |
| *dpr*A, *dal*A, *cil*B | SP_1266 | NP_345730.1 | 286 | SCRE_0874 | 280 | 75.7 |
| *ccl*A, *cil*C | SP_1808 | NP_346241.1 | 219 | SCRE_0451 | 212 | 57.1 |
| *ssb* | SP_1908 | YP_873936.1 | 131 | SCRE_1656 | 131 | 84.0 |
| *rec*A | SP_1940 | NP_346368.1 | 388 | SCRE_1796 | 386 | 88.1 |
| *cgl*G, *com*GG, *com*YG | SP_2047 | NP_346471.1 | 137 | SCRE_1683 | 100 | 43.0 |
| *cgl*D, *com*GD, *com*YD | SP_2050 | NP_346474.1 | 134 | SCRE_1686 | 127 | 56.7 |
| *cgl*C, *com*GC, *com*YC | SP_2051 | NP_346475.1 | 108 | SCRE_1687 | 102 | 66.7 |
| *cgl*B, *com*GB, *com*YB | SP_2052 | NP_346476.1 | 290 | SCRE_1688 | 289 | 68.9 |
| *cgl*A, *com*GA, *com*YA | SP_2053 | NP_346477.1 | 313 | SCRE_1689 | 313 | 75.7 |
| *com*FB | SP_2207 | NP_346618.1 | 220 | SCRE_0443 | 218 | 47.2 |
| *com*FA | SP_2208 | NP_346619.1 | 432 | SCRE_0442 | 428 | 70.3 |

* comEA, truncated in all SCP but full length genes are found in SCC, SI and SA. ^1^Locus tag for *S. pneumoniae* TIGR4 as identified by Peterson et al., 2004 [74]; ^2^HSP: Percent coverage of protein from SAG compared to best match in NCBI database using BlastP ; ^3^PID is equal to the percent aa identity for the best match using BlastP from NCBI
