# Supplementary material for: Phylogenetic relationship and virulence inference of Streptococcus Anginosus Group: curated annotation and whole-genome comparative analysis support distinct species designation
Source: BMC Genomics. 2013 Dec 17;14:895. doi: 10.1186/1471-2164-14-895 (PMC3897883; doi:10.1186/1471-2164-14-895)
Supplement: Additional file 17: Table S14 — SNPs found within chromosomal sequence for SCP C232 as compared to SCP C818. [file 1471-2164-14-895-S17.docx]

Additional file 17, Table S14: Single nucleotide polymorphisms (SNPs) found within chromosomal sequence for SCP C232 as compared to SCP C818.

| SNP Position | CDS/Intergenic | Gene Name | C232 Sequence | C818 Sequence | C232 Codon | C818 Codon | C232 Residue | C818 Residue | Predicted Product |
| --- | --- | --- | --- | --- | --- | --- | --- | --- | --- |
| 56206* | Intergenic |  | C | A |  |  |  |  |  |
| 111453 | Synonymous | adcA | A | C | GCA | GCC | A | A | Zinc-binding lipoprotein |
| 288491 | Synonymous |  | A | G | TCA | TCG | S | S | Putative transcriptional regulator |
| 303457 | Intergenic |  | G | T |  |  |  |  |  |
| 736634 | Non-synonymous | pta | C | T | ACC | ATC | T | I | Phosphate acetyl/butaryl transferase |
| 997157 | Non-synonymous |  | G | T | GAG | TAG | E | Stop | Putative ABC transporter; ATPase |
| 1217955 | Intergenic |  | (T) | (A) |  |  |  |  |  |
| 1299963 | Synonymous |  | (C) | (A) | (CGA) | (AGA) | R | R | Hypothetical protein |
| 1314972 | Non-synonymous | aroD | (G) | (T) | (GGA) | (TGA) | G | Stop | 3-dehydroquinate dehydratase, type I |
| 1412046 | Non-synonymous |  | (G) | (C) | (AGC) | (ACC) | S | T | Putative transcriptional repressor |
| 1543861 | Intergenic |  | T | G |  |  |  |  |  |
| 1704788 | Intergenic |  | G | A |  |  |  |  |  |
| 1704803 | Intergenic |  | G | T |  |  |  |  |  |
| 1731563 | Non-synonymous | comYD | (A) | (C) | (GAT) | (GCT) | D | A | Competence protein |
| 1741412 | Non-synonymous | rpoC | (G) | (A) | (GGA) | (GAA) | G | E | DNA-directed RNA polymerase, beta’ subunit |
| 1745683 | Non-synonymous | rpoB | (C) | (G) | (TCT) | (TGT) | S | C | DNA-directed RNA polymerase, beta’ subunit |
| 1745699 | Non-synonymous | rpoB | (A) | (C) | (AAC) | (CAC) | N | H | DNA-directed RNA polymerase, beta’ subunit |
| 1912615 | Non-synonymous | cbiQ | (G) | (T) | (AGT) | (ATT) | S | I | putative cobalt transport protein |

*SNP position is annotated in relation to *S. constellatus* subsp. *pharyngis* C232, and nucleotide composition refers to that of the positive strand unless the SNP lies in coding sequence of the opposite strand it is then shown as complement using brackets.
