# Supplementary material for: Phylogenetic relationship and virulence inference of Streptococcus Anginosus Group: curated annotation and whole-genome comparative analysis support distinct species designation
Source: BMC Genomics. 2013 Dec 17;14:895. doi: 10.1186/1471-2164-14-895 (PMC3897883; doi:10.1186/1471-2164-14-895)
Supplement: Additional file 18: Table S15 — Tandem-repeats and microsatellite differences identified between SCP C232 and SCP C818. [file 1471-2164-14-895-S18.docx]

Additional file 18, Table S15: Tandem-repeats and microsatellite differences identified between SCP C232 and SCP C818.

| Repeat start position | CDS | Repeat | C232 | C818 | Predicted product |
| --- | --- | --- | --- | --- | --- |
| 364593* | SCRE_0376 | ACCCTCAGCTATTCAG (complement) | 1X | 2X | transposase (truncated) |
| 429219 | SCRE_0436 | GAAGTTAAGCCAGAGGTTAAACCG (complement) | 5X | 12X | conserved hypothetical protein |
| 546213 | SCRE_0553 | AGTG | 1X | 2X | Type-I restriction enzyme (truncated) |
| 629218 | Intergenic | GTCAGACAGCTTGCCTGAAAAGAGATTCACTCCATCCGAGG | 3X | 4X |  |
| 1424629 | Intergenic | GCTCT | 31X | 35X |  |

*Repeat start position is annotated in relation to SCP C232, nucleotide positions refers to the positive strand unless the repeat is within a coding sequence and then complement is identified.
